# Empowering safety managers to champion the implementation of smoking cessation services in the construction industry: Protocol for a sequential multiple assignment randomized trial

**DOI:** 10.1371/journal.pone.0324717

**Published:** 2025-06-09

**Authors:** Taghrid Asfar, David J. Lee, Ramzi G. Salloum, Jennifer H. LeLaurin, Erin Kobetz, Nipesh Pradhananga, Roxana A. De Dios Despaux, Kathryn E. McCollister, Olusanya Oluwole, Laura Corbin, Jennifer Laine, Zoran Bursac

**Affiliations:** 1 Department of Public Health Sciences, University of Miami Miller School of Medicine, Miami, Florida, United States of America; 2 Sylvester Comprehensive Cancer Center, University of Miami Miller School of Medicine, Miami, Florida, United States of America; 3 Department of Health Outcomes and Policy, and Institute for Child Health Policy, College of Medicine, University of Florida, Gainesville, Florida, United States of America; 4 Engineering and Computing, Moss Department of Construction Management, Florida International University, Miami, Florida, United States of America; 5 Bureau of Tobacco Free Florida, Florida Department of Health, Tallahassee, Florida, United States of America; 6 Department of Biostatistics, Florida International University, Miami, Florida, United States of America; PLOS: Public Library of Science, UNITED KINGDOM OF GREAT BRITAIN AND NORTHERN IRELAND

## Abstract

US construction workers (CWs) have the highest cigarette smoking rate among all occupations (27.2% vs. 15%), yet the lowest coverage of workplace smoking cessation services (14% vs. 29%). This study aims to empower safety managers to implement smoking cessation services in the construction industry. Using participatory research methods, this study aims to: 1) Develop multilevel strategies (MLIs) to implement adaptive smoking cessation programs delivered by the safety manager on construction sites, and 2) conduct a cluster-randomized, hybrid type 1 effectiveness-implementation, 2-phase sequential multiple assignment randomized trial (SMART) to test the programs (ClinicalTrials.gov: NCT06098144). The MLIs include: 1) creating the outer setting (research investigators, stakeholders) and inner setting facilitation (companies’ advisory committee, study champion), 2) conducting observational field assessments of workflows, 3) training safety managers to deliver the intervention, and 4) conducting implementation process evaluations. In SMART, 32 construction sites within 8 companies with 608 CWs will be enrolled. In Phase 1, sites will be randomized to A1 (referral to Tobacco Quitline -TQL) or B1 (referral to TQL + nicotine replacement treatment (NRT) + 1 group behavioral counseling session). In Phase 2, responders who quit smoking at 3 months continue with the assessment only, while non-responders will be re-randomized to C (4 counseling sessions + NRT; A1 + C, B1 + C) or an extra dose of Phase 1 treatment (A2, B2). Participants will receive 4 follow-up assessments at 3, 6, 9, and 12 months. Primary outcomes are the effectiveness (12-month biomarker-confirmed prolonged abstinence) and cost-effectiveness (cost/quit, cost/quality-adjusted life-year) of A1 + A2 vs. B1 + B2 and A1 + C vs. B1 + C. The secondary outcome is the feasibility of the program’s implementation. We hypothesize that B1 + B2 will outperform A1 + A2, and B1 + C will outperform A1 + C. This project will generate novel scientific evidence on the effectiveness, cost-effectiveness, and implementation feasibility of smoking cessation programs in the construction industry.

## Introduction

Construction workers (CWs) in the United States (U.S.) have the highest smoking prevalence compared to other occupational groups and the general population (27.2% vs. 21.8% and 17%) [1,2]. Almost half of U.S. CWs (43%) are racial/ethnic minorities, with substantially lower hourly wages compared to White CWs ($16 vs. $21) [3]. CWs Cessation efforts are hindered by their mobility and low access to smoking cessation services [4–6]. Workplace smoking cessation programs are consistently recommended by authorities to reduce smoking among U.S. working adults [7]. These programs have been proven to be effective and cost-effective by reducing smoking-related diseases [2,8]. However, CWs have the lowest coverage by such programs among all occupations (14% vs. 29%) [9]. The limited availability of workplace cessation programs in the construction industry, combined with the high rates of cigarette smoking among workers, underscores a significant opportunity to implement workplace smoking cessation programs that could help reduce tobacco use and contain costs within the construction sector.

To date, only a few smoking cessation trials have been conducted among CWs [1,7,9], and all are solely focused on individual workers without considering organizational factors that might affect program implementation, such as leadership engagement, support, and costs [10–12]. To address this gap in research, this project aims to implement and test several adaptive smoking cessation programs with increasing intensity delivered by safety managers in the construction sector. This will be achieved by actively engaging leadership and evaluating costs. Findings will help create a more robust framework for successful smoking cessation initiatives that benefit both workers and the organization in the construction sector.

Expanding the safety manager’s role to implement a sustainable smoking cessation service in the construction industry aligns with the National Institute for Occupational Safety and Health’s Total Worker Health recommendations for integrating disease prevention in work-related safety and health [13]. Safety managers are responsible for the safety and well-being of CWs, are trusted by them, and have daily contact with them, which makes them optimal for delivering a sustainable smoking cessation treatment [14–16]. In addition, one of the best achievements in tobacco control has been the creation of the *Tobacco Quitlines* (TQLs) [17]. TQLs are free, available in Spanish/English, and allow flexible timing, which makes them suitable for our target population. While their efficacy and potential reach are extensively documented [18], relatively few smokers utilize them [19], and even modest increases in their reach could reduce smoking at the population level [20,21]. TQL usually provides two services: “proactive,” where the TQL counselor initiates the call with the smoker, and “reactive,” where the smoker initiates the call with the TQL. Compared to the reactive service, the proactive service doubles quit rates [18]. An option to improve the reach for proactive service is using the “fax referral service,” where the interventionist completes a form and faxes it to TQL. This allows the TQL counselor to call smokers to provide up to 4 phone counseling sessions, a free 2-week supply of NRT, and an outcome report to be sent back to the referring party within 2 weeks. In addition, proactive counseling in single-session face-to-face programs is effective in improving smoking cessation outcomes in the difficult-to-reach population [22,23]. Thus, given their availability, flexibility, and effectiveness, these two methods have a high potential for implementation in the construction sector.

Multilevel implementation strategies (MLIs) can simultaneously impact multiple contextual levels (e.g., individual, organization) to enhance health outcomes by creating a more efficient and coordinated delivery system [24–26]. Adaptive intervention design optimizes long-term cessation outcomes by personalizing treatment through decision rules that increase program intensity when individuals do not respond to the initial intervention. This method identifies the least resource-intensive program that still achieves acceptable results [27]. The Sequential Multiple Assignment Randomized Trial (SMART) is a multistage experimental design developed to optimize adaptive programs [28]. In Phase 1, patients are randomized to two active treatments. In Phase 2, patients who respond to the initial treatment continue that treatment, while non-responders are re-randomized to additional treatment (more intensive) [29]. Finally, the hybrid type 1 effectiveness-implementation trial design allows researchers to evaluate the implementation and effectiveness of the program simultaneously to reduce the time to uptake evidence-based interventions [30]. In this manuscript, we present the study protocol of a randomized clinical trial designed to expand the role of safety managers to implement a workplace smoking cessation program in the construction industry.

## Materials and methods

### Study design

#### Objectives.

This study aims to: 1) develop MLIs to integrate smoking cessation programs in the construction industry, and 2) conduct a hybrid type 1 effectiveness-implementation SMART to test the effectiveness, cost-effectiveness, and implementation feasibility of six adaptive smoking cessation programs with increasing intensity delivered onsite by the safety manager. We hypothesize that B1 + B2 will outperform A1 + A2, and B1 + C will outperform A1 + C. Recruitment in the study started on 4/10/2024 and will continue until 10/01/2027. Participants provided written informed consent that is documented and witnessed.

#### Design.

The University of Miami Institutional Review Board approved the study on July 13, 2023, under IRB number 20230549, classifying it as “Low Intervention Risk” due to its pragmatic design. The study is currently registered in the ClinicalTrials.gov registry with the identifier NCT06098144.

**Fig 1:**
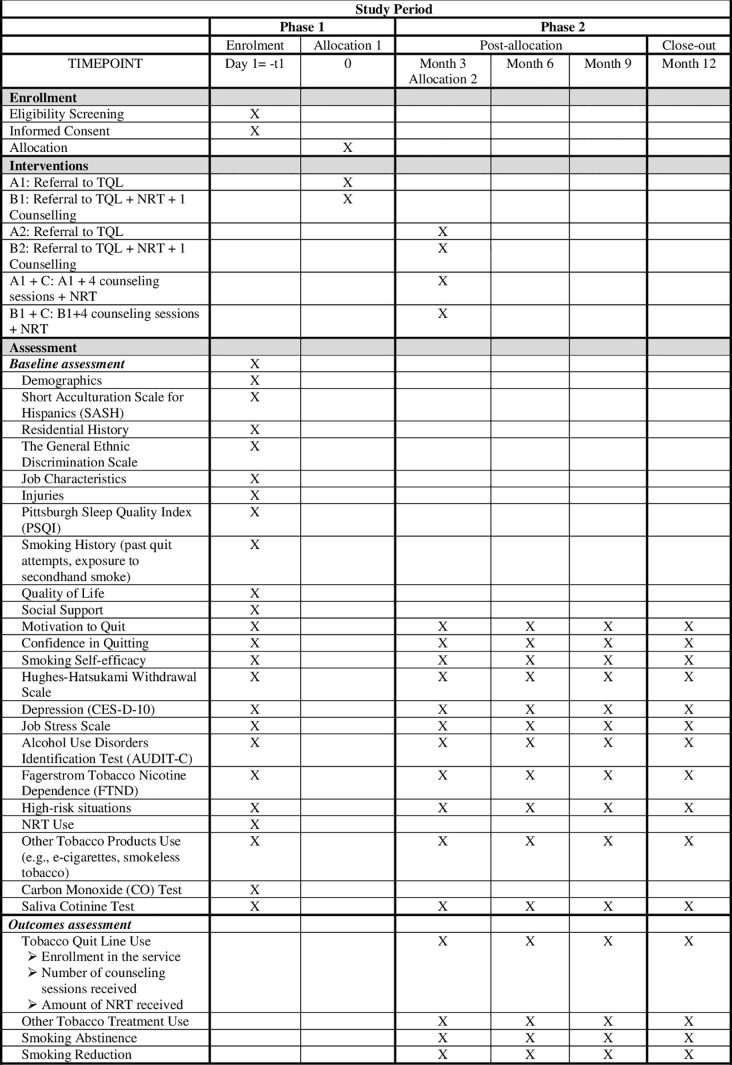
Study period.

**Fig 2:**
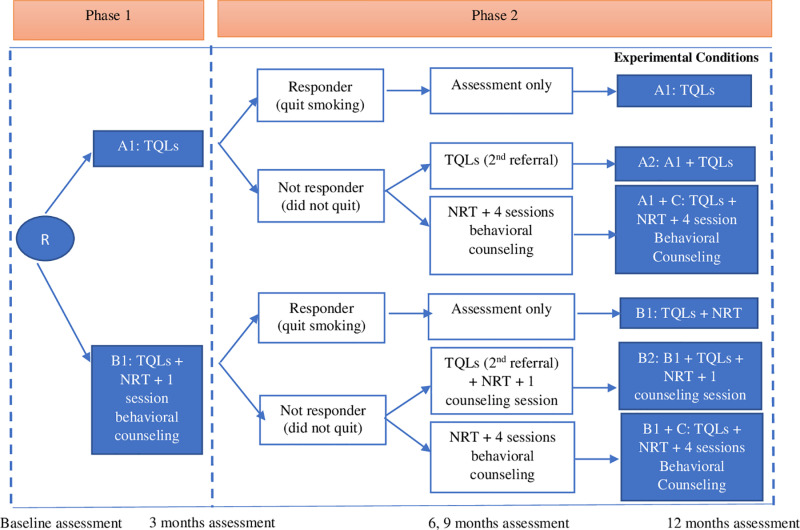
SMART design (Aim 2).

We will recruit 32 construction sites within 8 construction companies with 608 CWs to conduct 2-arm, cluster, hybrid type 1 effectiveness-implementation, 2-phase SMART to test the 6 adaptive programs with increasing intensity. The 6 programs are: A1) referral to Tobacco Quitline (TQL), B1) referral to TQL + nicotine replacement treatment (NRT) + 1 group behavioral counseling session with the safety manager, A1 + C) A1 + 4 counseling sessions + NRT, B1 + C) B1 + 4 counseling sessions + NRT, A2) A1 + TQL, and B2) B1 + TQL + NRT + 1 group behavioral counseling session. In Phase 1, construction sites will be randomly assigned into two conditions, A1 or B1. All participants in A1 and B1 will receive a 3-month assessment to determine their smoking status. Responders who quit smoking at the 3-month assessment will continue in the same treatment (A1, B1) in Phase 2. Non-responders who did not quit smoking will be re-randomized either to C (conditions A1 + C and B1 + C) or to an extra dose of the same treatment in Phase 1 (conditions A2, B2). In Phase 2, all participants will receive 3 follow-up assessments at 6, 9, and 12 months after enrollment (Fig 1 and Fig 2). Primary outcomes are the effectiveness (12-month biomarker-confirmed prolonged abstinence) and cost-effectiveness (cost/quit, cost/quality-adjusted life-year) of A1 + A2 vs. B1 + B2 and A1 + C vs. B1 + C. Secondary outcomes are the programs’ implementation feasibility (acceptability, barriers/facilitators).

#### Conceptual framework.

**Fig 3:**
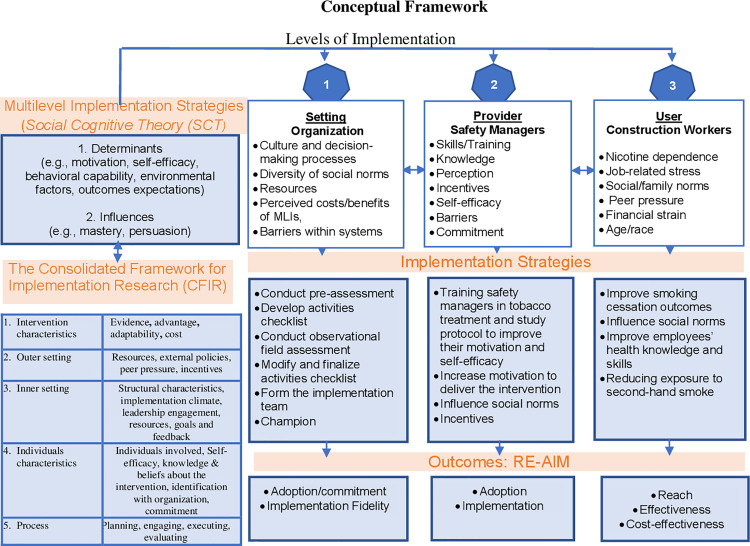
Conceptual framework.

The Consolidated Framework for Implementation Research (CFIR) guides the development of the MLIs [31]. The CFIR highlights five domains that influence the successful implementation of a new program into practice: 1) program characteristics (evidence strength and adaptability), 2) outer setting facilitation (needs and resources), 3) inner setting facilitation (implementation climate, communications), 4) characteristics of involved individuals (e.g., knowledge, beliefs); and 5) process implementation (e.g., planning, executing, evaluating) (Fig 3). The SMART study is guided by the RE-AIM (Reach, Effectiveness, Adoption, Implementation, Maintenance) framework, which assesses program outcomes across various settings and behavioral outcomes to enhance real-world integration [32–35].

### Setting

The study will be implemented in 36 construction sites within eight companies in South Florida. Three levels will be considered for implementing the program, including organization (e.g., culture, cost/benefits, barriers/facilitators), safety managers delivering the treatment (e.g., training, incentives, self-efficacy), and CWs (e.g., nicotine dependence, job-related stress, social/family norms, peer pressure, age, race). According to the CRRI, the implementation activities will include several steps (Table 1):

**Table 1:** Planned multi-level implementation strategies.

Strategies	Definition	Actors
**1.**Build a coalition to develop an inner support system (internal facilitation)	Each construction company will form an “Advisory Committee” to guide the development and implementation of the program within its work environment. The committee will have a bimonthly conference call with the study investigators during the project to discuss their implementation activities.	Each committee will include: A study “champion” who will play a liaison role between the company and the research staff. A representative for safety managers. A representative for CWs.
**2.**Provide external facilitation (the study investigators and research staff)	Provide ongoing consultation on program training and delivery and help problem-solve. Track all facilitation activities, report on implementation status and progress, and complete the implementation evaluation. Have a monthly videoconference with the company’s implementation advisory committees to discuss progress and improvement in implementation.	The study investigators, 2 Research Assistants and 2 graduate students with backgrounds in public health.
**3.**Assess for readiness and identify barriers and facilitators	The research team will conduct a pre-implementation assessment meeting with opinion leaders and safety managers to present the scientific evidence behind the program and its benefits to the organization. The meeting will also include a discussion on how the program should best be implemented to fit the organizational context and work environment and expected barriers and facilitators.	The study investigators, 2 Research Assistants, and 2 graduate students with backgrounds in public health.
**4.**Perform an observational field assessment of workflow on construction sites	The research team will shadow safety managers and conduct informal interviews to identify implementation barriers and facilitators. The assessment will provide perspective on the needs and assets of the existing workflow to minimize disruption and enhance program delivery and integrity.	The study investigators, 2 Research Assistants, and 2 graduate students with backgrounds in public health.
**5.**Develop an implementation activities checklist	The research team will create an implementation activity checklist and then meet with the company’s Advisory Committee to review and revise it based on their needs.	The study investigators, 2 Research Assistants, and 2 graduate students with backgrounds in public health.
**6.**Training safety managers to deliver the program	Safety managers will receive training in: 1) program delivery and fidelity, 2) study protocol, 3) human subjects protection, and 4) a 10-hour self-paced tobacco treatment online course, “Basic Skills for Working with Smokers.”	The study investigators.
**7.**Develop and organize a quality monitoring system	The research team will track and document all implementation activities in a time motion tracking log, assess the fidelity and intensity (quality and depth) of implementation, timeliness of task completion, and degree of engagement of key involved individuals throughout the process.	The study investigators, 2 Research Assistants, and 2 graduate students with backgrounds in public health.
**8.**Conduct implementation process evaluation	The research team will conduct 3 mixed-methods of quantitative and qualitative process evaluations (pre-, mid-, and post-implementation) to help improve implementation strategies.	The study investigators, 2 Research Assistants, and 2 graduate students with backgrounds in public health.

a) Program characteristics: The research team will meet with opinion leaders, safety managers, and representatives of CWs to present the scientific evidence behind the program and its benefits to the organization. The meeting will discuss how implementation can fit the organizational context, along with expected barriers and facilitators.
b) Outer setting facilitation: The research investigators will provide ongoing consultations regarding program training and delivery during the study. They will assist in problem-solving and offer continuous technical support for developing and implementing tools for data collection, quality monitoring, and tracking activities. This will include using a time-motion tracking log to evaluate the fidelity and intensity (quality and depth) of implementation [36], the timeliness of task completion, and the level of engagement of the involved individuals.
c) Inner setting facilitation: Each construction company will form an “advisory committee” to guide the program’s implementation. The committee will include a study “champion” who will be a liaison between the company and the research staff and facilitate and oversee all on-site implementation activities, as well as representatives for safety managers who will deliver the program and CWs. Investigators and advisory committees will hold monthly meetings to ensure effective program implementation.
d) Characteristics of involved individuals. Before implementation, researchers will collect surveys from leaders, safety managers, and workers to identify key factors affecting the program.

e) Process evaluation: The research team will conduct an observational field assessment of construction site workflows by shadowing safety managers and conducting informal interviews to identify barriers and facilitators. This will inform the development of a tailored implementation checklist to enhance program delivery. In addition, 3 process evaluations (pre-, mid-, and post-implementation), including interviews and surveys, will be conducted to improve implementation, analyze primary outcomes (e.g., cessation rate), and provide recommendations for future efforts.
f) Training the safety manager: Each company will be asked to identify the safety manager who will be delivering the intervention. Upon their agreement, safety managers who accept to participate in the study will receive one-day training by study investigators in the following: (a) study protocol, (b) human subjects protection, (c) clinical documentation using Redcap Software (a free cloud-based tool from the University of Michigan), (d) program fidelity, and (e) practical experience in program delivery with standardized encounters using an actor simulating a worker enrolled in the study. They will also complete three hours of online training from the Florida Area Health Education Centers Network to deliver effective tobacco use treatment.

### Recruitment and study participants

Our targeted accrual goal is 12–18 company leaders (≈1–2/company), 18–20 safety managers (≈2/company), and 608 workers who currently smoke and are willing to quit smoking. The company leaders and safety managers will participate in the program’s implementation assessment (key informant interviews, surveys), and workers will participate in the SMART study to receive the smoking cessation interventions. Inclusion criteria for company leaders are to be ≥ 18 years old and involved in decision-making in the company. Inclusion criteria for safety managers are to be ≥ 18 years old, non-cigarette smokers, willing to receive tobacco treatment training, willing to deliver the treatment to workers, and not planning to leave the company in the next year to deliver the intervention. Inclusion criteria for CWs are: being a construction worker in the participating company, being an adult aged ≥18 years old, having smoked an average of ≥5 cigarettes/day in the past year, being interested in making a serious quit attempt in the next 30 days, own a telephone with a plan to keep it active for the next 12 months, and not planning to leave the company in the next 6 months. Exclusion criteria for CWs are the inability to provide the consent form and women who are pregnant or nursing (lactating) or of childbearing age planning to become pregnant. CWs who use other tobacco products (e.g., smokeless tobacco, e-cigarettes) will be included in the study to maximize generalizability. Participants with contraindications to NRT can enroll in the study but will not receive NRT [17].

### Procedures and data collection

The safety manager will introduce the study and invite CWs to participate during their regular morning safety briefings. Interested participants will meet with one of our research assistants (RAs) during their breakfast/lunch break to be screened and, if eligible, provide written informed consent. The RAs are trained in public health research, study protocol, and human subject protection. Then, participants will complete a 15–20 minute baseline assessment after enrollment, followed by the treatment according to their site randomization the next day to minimize workflow interruption. Given the large population of Hispanic/Latino CWs in Florida [30], all study materials will be available in English and Spanish and administered by bilingual (RAs, safety managers) staff based on participant preference. Participants will receive follow-up phone calls at 3, 6, 9, and 12 months to assess smoking status, use of additional NRT or cessation drugs, other tobacco product use, and contact with the safety manager and TQL. Biochemical validation of smoking status using salivary cotinine analysis will be performed on a random sub-sample of participants reporting prolonged abstinence at the 3 and 12-month assessments within 50 miles of the institution. We will use the disconfirmation rates from this sample to estimate adjusted smoking rates for the entire sample. Saliva samples will be collected using NicoTests®, which detects cotinine in oral fluid/saliva at cut-off concentrations of 30 ng/mL (< 30 indicates abstinence).

### Interventions

#### Rational.

The smoking cessation programs were chosen based on evidence of their feasibility, acceptability, and potential efficacy from our pilot study [12], evidence on their effectiveness and cost-effectiveness in the general population [17,37]), company leaders’ interest [16], their appropriateness to CWs’ work and life circumstances [38], and their potential for scalability and dissemination [12]. TQLs are free to all Floridians, available in Spanish/English, and allow flexible timing, which makes them suitable for our target population [18]. In addition, evidence suggests that single-session face-to-face programs are particularly effective in low-income and minority populations that are difficult to reach [22,23]. Finally, several meta-analyses indicated that increasing the intensity (amount, period) of the behavioral support as an adjunct to NRT is likely to increase the chance of success in quitting by 10% to 25% compared with less or no support [39–41]. These programs are particularly promising for heavily dependent smokers, such as our target population [17]. Based on this evidence, and given their flexibility, effectiveness, and potential for scalability, these three evidence-based interventions have a high potential for implementation and dissemination in the construction sector.

#### Interventions description.

This study will test 6 adaptive interventions: A1 and B1 in Phase 1, and A2, B2, A1 + C, and B1 + C in Phase 2. Below, we describe the intervention in each group.

**Group A1** This group includes referrals to the TQL using the “fax referral form.” Participants will be informed that the TQL is free, and the TQL counselor will provide up to 4 phone counseling sessions to devise a specific plan to quit smoking and arrange the delivery of up to 12 weeks of free NRT. Participants will be advised to request nicotine gums instead of patches to accommodate their job circumstances (excessive heat).

**Group B1** In addition to A1, CWs in B1 will receive 6 weeks of NRT, a brief (15–20 min) in-person behavioral group counseling session, and two follow-ups (in-person or by phone) (Table 2) [17]). The behavioral counseling session has been developed based on the social cognitive theory [42,43]), previous formative work [44], and cessation literature [17,45–48]). The session will discuss preparing to quit, coping with stress, getting social support, the “5 A’s” for preventing relapse (Avoid, Alter, Alternatives, Anticipate, and Active), and proper use of NRT [17]. The first follow-up occurs 1 day before the quit date to remind participants about their quit date and provide extra support. The second follow-up occurs 2 weeks after the quit date to discuss progress and skills to prevent relapse and help reengage the participant in another quit attempt if they have lapsed [17].

**Table 2:** Intervention B1 and B2.

Date	Group session	Goal
Day 1	Session 1	Provide intervention in a group session (the safety manager): • Set a goal for a 30% smoking reduction in week 1. • Set a quit date. • Prepare to quit (quitting ritual). • Learn to cope with job stress (e.g., deep breathing, reducing caffeine intake). • Get social support (co-workers, family) • Avoid high-risk situations (5 A’s: Avoid, Alter, Alternatives, Anticipate, and Active). Refer participants to the Florida Tobacco Quitline (the research staff). Distribute 6 weeks of NRT.
Day 14: 1 day before quit date	Follow-up (1)	Review progress in smoking reduction Confirm quit date Review quitting plan Provide support and praise successes Prevent relapse (short term) Complete call 1 Assessment Questionnaire (the research staff).
Day 30: 2 weeks after quit date	Follow-up (2)	Review progress Provide support and praise successes Prevent relapse (long term) Complete call 2 Assessment Questionnaire (the research staff).

**Group A1 ± C and B1 ± C**: Participants who do not quit in Phase 1 and are re-randomized to C in Phase 2 will receive 6 weeks free supply of NRT from the research team, 4 (20-min) group behavioral

counseling sessions, and two follow-ups (in-person or by phone) (Table 3). Session 1 (2 weeks before the quit date) discusses reasons to quit, preparing to quit, coping with negative affect and job stress, and the “5 A’s.” Session 2 (on the quit date) focuses on short-term relapse prevention training, surviving the first few days as a nonsmoker, challenges in previous quit attempts, and managing nicotine withdrawal. Session 3 (2 weeks after the quit date) determines if participants quit; if not, we will record reasons, reset the quit date, and review motivating images. Session 4 (4 weeks after the quit date) focuses on long-term relapse (4 rules for limiting access to cigarettes, personalized relapse

**Table 3:** Intervention “C” schedule and content.

Date	Sessions	Goals
Day 1	Session (1)	Set a quit date & prepare to quit Review challenges during previous quit attempt in phase 1 Avoid high risk situations (5 A’s) Coping with negative affect and job stress Getting social support (co-workers, family) Distribute 8 weeks of NRT and review proper use of nicotine patch
Day 15: on quit date	Session (2)	Determine if the participant quits Discuss how to survive the first few days as a nonsmoker Discuss short-term relapse prevention training (coping strategies for high-risk situations) Managing nicotine withdrawal Discuss “be good to yourself” Review proper use of nicotine patch
Day 30: 2 weeks after the quit date	Session (3)	Determine if the participant quits. If not, process the reasons and reset the quit date. Reinforce successes and problem-solving strategies for dealing with difficult situations. Reinforce patch use Review motivating images to quit smoking
Day 45: 4 weeks after the quit date	Session (4)	Determine if the participant quits. If not, process the reasons and reset the quit date. Reinforce successes and problem-solve difficult situations. Reinforce patch use. Discuss negative thoughts (examples and strategies for dealing with them). Discuss long-term relapse prevention and the four rules for limiting access to cigarettes
Day 60: 6 weeks after the quit date	Follow-up (1)	Review progress Provide support and praise successes Prevent relapse
Day 75: 8 weeks after the quit date	Follow-up (2)	Review progress Provide support and praise successes Preventing long-term relapse

plan) and negative thoughts. NRT use and adherence, or side effect issues, will be discussed in every session. In the first (6 weeks after the quit date) and second follow-ups (8 weeks after the quit date), we will discuss progress and skills to prevent relapse and help reengage another quit attempt if they have lapsed.

**Group A2 and B2** Participants who do not quit in Phase 1 and are re-randomized to their original treatment in Phase 2 will receive the same treatment in A1 and B1. The focus will be on utilizing the insights gained from Phase 1 and implementing effective problem-solving strategies to address the high-risk situations contributing to relapse.

All participants will receive information about the incremental risks of smoking on CWs, given their exposure to occupational hazards, advice to quitting all tobacco products, and self-help materials summarizing their program in their preferred language. The initial dose of Nicoderm™ gums (2 mg, 4 mg) will be established based on cigarette consumption [17]. Participants will receive instructions on proper gum use and potential side effects. NRT will be kept in a temperature-controlled field office and monitored by the site safety manager.

#### Strategies to improve adherence to interventions.

The study team has an excellent history of retaining clinical trial participants among the target population [12]. Active communication and consideration of safety managers and CWs’ time when scheduling research activities will be maintained during the study. Compliance and retention will be maximized by: (a) holding two annual meetings with the companies’ advisory committee to review progress, (b) coordinating with safety managers to schedule site visits for enrolling participants and completing the required assessments during workers’ breaks, (c) documenting all safety managers’ activities in RedCap, (d) conducting a monthly meeting with safety managers to address problems and discuss individual cases, and (e) establishing rapport and maintain active communication with participating CWs throughout the study.

### Measures

#### Baseline assessment.

The baseline assessment includes sociodemographic (age, sex, race/ethnicity, income, education), job characteristics, smoking history, nicotine dependence [49], stages of change [50], quit ladder [51], self-efficacy [52], depression (CES-D-10) [53], job stress [54], ASSIST for alcohol and substance use assessment [55], social support [56], exposure to secondhand smoke, and exhaled carbon monoxide (Table 4) [57].

**Table 4:** Assessments and Measures.

	Phase 1			Phase 2			
Instruments	Baseline (A1 & B1)	Follow-up 1 and 2 (B1)	3-month follow-up (A1 & B1)	Follow-up 1 and 2 (B2)	2 Intra-treatment assessments (A1 + C & B1 + C)	End-of-Treatment assessment (A1 + C & B1 + C)	6, 9 & 12 months follow-ups (All groups)
Contact Information	X						
Demographics	X						
Short Acculturation Scale for Hispanics (SASH)	X						
Residential History	X						
The General Ethnic Discrimination Scale	X						
Job Characteristics	X						
Injuries	X						
Pittsburgh Sleep Quality Index (PSQI)	X						
Smoking History (past quit attempts, exposure to secondhand smoke)	X						
Quality of Life	X						
Social Support	X	X		X			
Motivation to Quit	X	X	X	X			X
Confidence in Quitting	X	X	X	X			X
Smoking Self-efficacy	X		X				X
Hughes-Hatsukami Withdrawal Scale	X		X				X
Depression (CES-D-10)	X		X				X
Job Stress Scale	X		X				X
Alcohol Use Disorders Identification Test (AUDIT-C)	X		X				X
Fagerstrom Tobacco Nicotine Dependence (FTND)	X		X				X
Climate Questionnaire			X (only B1)			X	
High-risk situations		X (only follow-up 2)		X (only follow-up 2)	X		
Managing nicotine withdrawal		X (only follow-up 2)		X (only follow-up 2)	X		
Other Tobacco Products Use (e.g., e-cigarettes, smokeless tobacco)	X	X	X	X	X	X	X
NRT Use	X	X	X	X	X	X	X
Contact the Safety Manager			X			X	
Tobacco Quit Line Use **•** Enrollment in the service **•** Number of counseling sessions received **•** Amount of NRT received		X	X	X	X	X	X
Other Tobacco Treatment Use			X			X	X
Smoking Abstinence			X		X	X	X
Smoking Reduction			X		X	X	X
Carbon Monoxide (CO) Test	X				X		
Saliva Cotinine Test	X		X			X	X
*Phase 1: Follow-up 1 (B1) occurs 1 day before quit date* *Phase 1: Follow-up 2 (B1) occurs 2 weeks after quit date* *Phase 2: Follow-up 1 (B2) occurs 1 day before quit date* *Phase 2: Follow-up 2 (B2) occurs 2 weeks after quit date* *Phase 2: Intra-treatment assessments (A1 + C & B1 + C) occur during session 3 and session 4* *Phase 2: End-of-Treatment assessment (A1 + C & B1 + C) occurs during the 2*^*nd*^ *follow-up (8 weeks after the quit date)*							

#### Follow-up assessment at 3, 6, 9, and 12 months.

These assessments will evaluate smoking status [58], concomitant smoking and NRT use, use of other cessation drugs and tobacco products, number/time of total contact with the safety manager and TQL, the Minnesota the Minnesota Withdrawal Scale [59], and the Questionnaire of Smoking Urges-Brief Scale [60].

### Outcomes.

#### Primary outcomes.

Primary outcomes are the programs’ effectiveness (12-month validated prolonged abstinence) and cost-effectiveness (cost/quit, cost/quality-adjusted life-year) for A1 + A2 vs. B1 + B2 and A1 + C vs. B1 + C (Table 5). Prolonged abstinence is defined as self-reported no smoking, not even a puff, after a grace period of two weeks after the quit date confirmed by a saliva cotinine level of < 30 ng/mL [61]. Participants who quit cigarettes but switch to alternative nicotine products (e.g., NRT, smokeless tobacco) will be considered not abstinent according to guidelines [62]. A secondary outcome of “harm reduction” for those who exclusively switch to e-cigarettes will also be collected. For cost-effectiveness, we will utilize a standardized cost data collection survey based on the Drug Abuse Treatment Cost Analysis Program (DATCAP) [63]. Program costs include standard resource categories (e.g., personnel, advertisement, NRT, educational materials). Personnel costs are based on detailed time logs from study personnel that track time spent on research and program activities. Advertisement, NRT, and educational material costs are based on invoices and allocated according to the number of participants in each program. Research costs will be excluded from cost calculations to inform real-world program applications.

**Table 5:** SMART study outcomes.

Primary outcomes	Definition	Data Sources & Tools
Effectiveness	Biomarker-confirmed prolonged abstinence at 1-year follow-up	Smoking status at 3-, 6-, 9-, and 12-month follow-up assessment
Cost-effectiveness	Incremental cost per quit Incremental cost per QALY	Start-up & implementation costs Intervention & participant costs The EuroQol (EQ-5D)
**Secondary outcomes**		
Implementation feasibility	Adaptability (leaders, safety manager, workers) Suitability for the work culture Relevance to workers’ needs Relevance to leadership and staff culture Resources and incentives The Tobacco Quitline enrollment rates Intervention fidelity Reach	Tracking implementation Organizational Readiness to Change Assessment (ORCA)^1^ Surveys (safety managers, construction workers) Key informant interviews (leaders, safety managers) The Tobacco Quitline outcome report Characteristics of smokers who agree to participate versus those who decline Workers’ dropout rate in each program Workers’ enrolment rates (number invited/enrolled) Safety managers’ enrolment rates (number invited/enrolled) Implementation Checklist Intervention fidelity checklist
Implementation acceptability	Program adoption Construction workers’ satisfaction Construction workers’ attitude and perception about the intervention Safety managers’ attitude and perception about the intervention	Surveys (safety managers, construction workers) The Client Satisfaction Questionnaire The proportion of CWs who are screened, enrolled, and engaged in the treatment (adherence to treatment) The proportion of safety managers who are providing the treatment
Barriers and facilities	Organization commitment Providers (safety managers) and recipients (construction workers) perception Marketing efforts Resources needed to maintain intervention Adaptations needed to integrate the intervention	Organizational Readiness to Change Assessment (ORCA) Client Satisfaction Questionnaire^2^ Implementation Checklist Surveys (safety managers, CWs) Key informant interviews (leaders, safety managers) Implementation Checklist
Potential sustainability	Expectation about the program sustainability	The Intervention Sustainability Assessment Tool (PSAT)^3^ Surveys (safety managers, CWs) Key informant interviews (leaders, safety managers)

^1^Helfrich, C.D., et al., Organizational readiness to change assessment (ORCA): development of an instrument based on the Promoting Action on Research in Health Services (PARIHS) framework. Implementation Science, 2009. 4(1): p. 38.

^2^Attkisson, C.C. and T.K. Greenfield, Client Satisfaction Questionnaire-8 and Service Satisfaction Scale-30. 1994.

^3^Luke DA, Calhoun A, Robichaux CB, Elliott MB, Moreland-Russell S. Peer reviewed: the program sustainability assessment tool: a new instrument for public health programs. Preventing Chronic Disease. 2014;11.

#### Secondary outcomes.

Secondary outcomes are the programs’ implementation feasibility, acceptability, barriers and facilitators, and sustainability potential. Three evaluations (pre-, mid-, and post-implementation) among company leaders, safety managers, and CWs will be conducted (Table 5). Each evaluation involves: (a) Surveys conducted among participating leaders, safety managers, and CWs (n ≈ 36; randomly selected from SMART) will include the Organizational Readiness to Change Assessment (ORCA) [64], TQL enrollment rates [65], implementation acceptability, CWs

satisfaction using the Client Satisfaction Questionnaire [66], safety managers’ training, incentives,

and treatment delivery, self-efficacy, and behavior capabilities [65]), and implementation barriers; and (b) In-depth key-informant interviews conducted with leaders and safety managers. The interview guide includes a short series of closed-ended questions (e.g., demographics) and open-ended questions that focus on perceptions regarding evidence, inner/outer context, barriers and facilitators of implementation, program sustainability [67], and suggestions for implementation improvement.

### Randomization

In Phase 1, site-level randomization in a 1:1 ratio to A1 and B1 is conducted using stratified random sampling based on company and site size. Smaller companies will be grouped into the same stratum to ensure sufficient sites for stratification by size. In Phase 2, individual-level stratified randomization by site and by the treatment in Phase 1 will be conducted in a 1:1 ratio. Randomization will be done using computer-generated random permuted blocks with random block sizes to ensure balanced allocation over time. All randomization sequences will be generated by a study statistician, who will not interact with companies or CWs, using computer-generated random permuted blocks with random block sizes to eliminate bias and ensure balanced allocation over time.

In Phase 1, the research team will create the allocation sequence and communicate the allocation results to the safety managers. The safety managers will then inform the workers about the study. Following this, the research staff will enroll participants and assign them to the respective interventions. A similar procedure will be followed in Phase 2.

### Blinding

This study is a single-blind trial. Since the intervention is behavioral, the researchers and safety managers administering it will clearly know which treatment each participant receives. However, participants will be blinded to their allocation and not informed of their study conditions. They will be informed that they will receive a tobacco cessation intervention and NRT delivered by their safety manager. Outcome assessors and data analysts are not blinded but are not involved in the treatment.

### Data collection, management, and monitoring

Two authorized research assistants will enter data into RedCap, an online data collection platform. Our data analyst will periodically manage and check the data for accuracy. All data (questionnaires and biomarkers) will be checked for out-of-range values and normality. If any spurious data (outliers) were identified, we will fit nonparametric statistical models to assess the robustness of our primary parametric data analysis. All analyses will be performed with SAS/STATv15.2 and R, and significance will be set at the two-tailed alpha level of 0.05 within the scope of all available statistical and clinical evidence.

To ensure standardization of program content and delivery, we will use standardized training, treatment manuals, and procedures [45, 68–70]. Safety managers will use a checklist to document treatment delivery in each session. Participants will complete a brief questionnaire at the end of treatment to assess learning. Standard procedures will be used for the study protocol and data management. The research team will provide ongoing training and supervision for safety managers and RAs through biweekly meetings and process reviews. Additionally, 10% of counseling sessions will be audiotaped for review and feedback.

We will collect personal information about potential and enrolled participants through informed consent forms and questionnaires. We will ensure that all personal information is securely stored and accessed only by authorized personnel. We will share personal information only with the research team and regulatory agencies, and we will do so through password-protected access or encryption to ensure confidentiality.

### Sample size calculation and justification

Our power estimates are based on detecting a difference between A1 + A2 and B1 + B2, and between A1 + C and B1 + C on biomarker-confirmed prolonged abstinence at 12-month assessment using unadjusted proportions. We assume nominal values for the Type I and II error rates (i.e., 5% and 20%, respectively; two-tailed) and based power on 12-month biomarker-confirmed cessation rates. Our pilot study yielded quit rates of 20.3% in A1 and 27.7% in B1 at 6-month [12]. Relevant to C, a meta-analysis of 47 trials has shown that increasing the amount of behavioral support as an adjunct to pharmacotherapy for smoking cessation increased the chance of success in quitting by 10% to 25% [41]. For the first phase of the SMART, we assume a 40% TQL enrollment rate in A1 and a 60% TQL enrollment rate in B1 [12]. For the second phase, we assume a 12-month biochemical-verified abstinence rate of 15% in A1 + A2, 28% in B1 + B2, 30% in A1 + C, and 45% in B1 + C [12,39–41]. Within 8 companies, construction sites (n = 32; 4 sites/company) will be randomized to A1 or B1 initially (16 sites/group), each containing 15 participants for a total of 480. This design and sample size achieve a power of 80% to detect an increase in abstinence of 13% between A1 + A2 and B1 + B2 and 15% between A1 + C and B1 + C. These estimations assume that a chi-square test from a generalized estimating equations (GEE) analysis or a logistic regression model is used at an alpha level 0.05. Missing values are assumed to occur at random. The autocorrelation matrix of the outcome responses within a cluster is assumed to match compound symmetry structure with an intraclass correlation coefficient of 0.01. To account for a 20% loss for attrition, as observed in our pilot study, we will enroll 608 participants.

### Statistical analysis plan

The SMART provides the opportunity to test multiple hypotheses [71,72]. Our primary hypotheses address the capability of the specific programs in each phase to extend effectiveness beyond the preceding phase: 1) for static regimens, is B1 + B2 better than A1 + A2 at 12 months? and 2) for dynamic regimens, is B1 + C better than A1 + C at 12-month? Secondary analyses will compare embedded dynamic treatment regimens.

#### Program effectiveness.

Our primary effectiveness analyses will be performed using GEE to account for clustering sites within construction companies and participants within the site [73,74]. We will use robust standard errors for statistical inference, incorporating appropriate upward covariance adjustment to correct these estimates’ downward bias when the number of clusters is limited [75]. We will compare 12-month abstinence among participants assigned to the respective treatments by the designated follow-up times by fitting the weighted GEE model with repeated measures to estimate the group, time, and group-by-time interaction contrasts. Observations will be weighted based on their inverse probability of receiving the program due to imbalance by design since only ½ of the initial non-responders remain on the initial program. Separate analyses will be applied to compare dynamic treatment in Phase 2. We will use an inverse probability of program-weighted, piecewise generalized linear estimating equations and robust standard errors to estimate the immediate and sustained effects of treatment “C” for non-responders (condition A1 + C compared to B1 + C) on 12-month abstinence. To compare dynamic strategies appropriately, we will include both participants who responded at the initial follow-up and remained in the original treatment (conditions A1 + A2 or B1 + B2) and those who did not respond and were randomized to step up “C.” Each group will be weighted by the inverse probability of receiving the program path followed. Covariates include two time variables denoting the number of months spent in each phase: a first-phase program indicator and a second-phase program indicator. Interactions between time in the first phase and first phase program and time in the second phase and first phase program, second phase treatment, and the interaction between the first and second phase program are also included [76]. The marginal longitudinal piecewise model allows for trajectories to change at each decision point/phase, with all individuals in the population offered each embedded treatment regimen. An exchangeable working correlation matrix will be used, and bootstrapped standard errors will be calculated. A sensitivity analysis will be conducted by stratifying participants who miss more than two scheduled sessions and do not re-schedule. At each phase, GEE will be applied to a binary outcome model. It will include the company as a site-level factor and participants’ (CWs) level covariates as predictor variables to control for baseline imbalances when site-level randomization is used [77]. Other potential covariates (all fixed effects) that may be included in a model include nicotine dependence, job stress, self-efficacy, demographics (e.g., age, race), NRT side effects, use of other tobacco products or programs that may have influenced their abstinence rates, and program adherence.

Evaluating multiple hypotheses under the SMART design has the potential to inflate the study-wise Type 1 error [78]. To address this issue, we will designate effectiveness and cost-effectiveness as co-primary outcomes and consider all other analyses as secondary. Because our hypothesis tests for the six programs address distinct questions, we will apply a Bonferroni-Holm multiple comparison adjustment to account for the 2 co-primary outcomes when evaluating each program, but will not perform additional multiple-comparison adjustments for the six programs [78]. Secondary GEE analyses with a binary outcome model will also be used for exclusively switching to e-cigarettes after quitting cigarettes [62].

#### Program cost-effectiveness.

The cost-effectiveness analysis will follow recognized standards and will track start-up and program implementation resources for each program [79]. The primary cost-effectiveness analysis will be performed from the construction sector perspective. Secondary analyses will include both program and participant costs. In the secondary analysis, participant costs will include time missed from work to participate in the program. For greater generalizability of results, we will use a national hourly wage rate to determine the economic value of time for all participants. The cost-effectiveness analysis will include incremental cost/quit, and per QALYs [80,81]. Using either program start-up plus implementation costs (primary analysis) or program + participant costs (secondary analysis), we will determine the incremental cost-effectiveness of program B1 + B2 relative to A1 + A2 and B1 + C relative to A1 + C. The generic formula for the incremental cost-effectiveness ratio is: C_E_–C_C_/ E_E_–E_C _=IC _EC_/ IE_EC =_ ICER_E vs. C_; where C_E_ = average costs for an experimental group; C_C_ = average costs for a control condition; E_E_ and E_C_ are the measured effectiveness of the experimental and control conditions (abstinence, QALYs), IC and IE are incremental cost and incremental effectiveness for the experimental condition relative to control, and ICER is the incremental cost-effectiveness ratio for the experimental condition relative to control. For this study, the ICER will express the incremental cost per quit (12-month prolonged abstinence) and incremental cost per QALYs gained in program B1 + B2 vs A1 + A2, and B1 + C vs A1 + C. Sensitivity analyses will be conducted to explore potential sources of uncertainty in the resource units or unit costs and will develop confidence intervals around the cost-effectiveness ratios using Monte-Carlo simulation techniques [82,83]. We also will examine cost-effectiveness in terms of health-related quality of life (HRQoL) by calculating QALYs gained in each study condition over the course of the program [84].

#### Program implementation feasibility.

The program’s implementation feasibility is a secondary outcome that includes the program’s acceptability, barriers and facilitators, and potential for sustainability. We will conduct three evaluations (pre-, mid-, and post-implementation) among company leaders, safety managers, and CWs will be conducted (Table 5). Each evaluation involves:

a) Surveys conducted among participating leaders, safety managers, and CWs (n ≈ 36; randomly selected from SMART) will include the Organizational Readiness to Change Assessment (ORCA) [64], TQL enrollment rates [65], implementation acceptability, CWs satisfaction using the Client Satisfaction Questionnaire [66], safety managers’ training, incentives, and treatment delivery, self-efficacy, and behavior capabilities [65], and implementation barriers.
b) In-depth key-informant interviews conducted with leaders and safety managers. The interview guide includes a short series of closed-ended questions (e.g., demographics) and open-ended questions that focus on perceptions regarding evidence, inner/outer context, barriers and facilitators of implementation, program sustainability [67], and suggestions for implementation improvement.

### Safety and adverse event reporting

Severe adverse events (SAEs) will be reported to the IRB at the University of Miami within 24 hours after their detection. Relationships with the study medications will be evaluated, considering the opinion of the personnel notifying them of the adverse event and the safety information available for each active antibiotic permitted. A periodic reconciliation of safety data will be performed to ensure all the eCRF-gathered information is appropriately communicated to the competent authorities.

### Ethical and regulatory considerations

The University of Miami Institutional Review Board (IRB) approved the study on July 13, 2023, under IRB number 20230549, classifying it as “Low Intervention Risk” due to its pragmatic design. The last IRB modification was approved on March 3, 2025 (MOD00019563). The study is currently registered in the ClinicalTrials.gov registry with the identifier NCT06098144.

Principles of the Declaration of Helsinki are considered, and all participants must sign an informed consent form before any procedure is done. Accordingly, written informed consent will be obtained from all participants before randomization and intervention. Participants will receive $100 in incentives ($20x5 assessments). Safety managers will receive a $500 gift card for participating in the study. In the implementation process evaluation, CWs (n ≈ 36; randomly selected from SMART) and safety managers (n ≈ 20) will receive an additional $60 for completing 3 surveys ($20 each). Participating leaders (n ≈ 12) in the key informant interviews will receive university-themed memorabilia (t-shirt, cup, bag).

Follow-up reports on recruitment and data safety updates will be sent to the UM IRB. Data protection legislation is considered for any data treatment in the study. Access to study data will be restricted to investigators until the database is completely locked, analyzed, and published. Results will be published according to CONSORT standards [85].

Although the UM Data Safety Monitoring Committee (DSMC) does provide oversight to social-behavioral studies, this trial does not require or benefit from the UM DSMC for two reasons: 1) the risk level is low (there is no risk to subjects in terms of the therapeutic interventional treatment), and 2) endpoints are not considered to be highly favorable or unfavorable result, or even a finding of futility, at an interim analysis might ethically require termination of the study before its planned completion.

## Discussion

This project is committed to eliminating tobacco-related health disparities among CWs by developing rigorous multi-level implementation strategies and testing adaptive smoking cessation programs delivered by the safety managers within the construction sector. This program will be constructed through a strong collaboration with construction company leadership, safety managers, and construction workers, ensuring its effectiveness and relevance. Expanding the safety manager role in delivering the program is an innovative approach to engage and build trust with a highly mobile and understudied workforce. Furthermore, utilizing the SMART design to test several adaptive smoking cessation programs with increasing intensity to generate needed data on the optimal treatment algorithm for implementation in the construction sector in terms of effectiveness and cost-effectiveness is highly innovative. This will also determine whether progressively adding more cessation resources increases effectiveness over and above implementing less resource-intensive approaches. The use of a hybrid type 1 effectiveness-implementation design promises to speed up the process of evidence generation and translation. Finally, the study employs many core elements of pragmatic trials to maximize generalizability [86], including using minimal exclusion criteria, enrolling a diverse set of CWs, and using intent-to-treat analyses [87–89]. Overall, this design promises to speed up program uptake in real-world settings, improve understanding of key organizational factors for long-term use, and reduce costs by streamlining and combining elements of the traditional step-wise progression of research [30].

There are several challenges with this study. We may encounter challenges in recruiting construction companies and workers. To mitigate this risk, commitments from eight construction companies were secured before receiving the funding. If needed, more companies will be recruited. The study team has a strong history of successful collaboration with the construction industry, ensuring successful project outcomes and innovative solutions. Busy work schedules may affect safety managers’ commitment to the study, but the research team will provide ongoing communication and support. Low fidelity poses another potential challenge. Therefore, additional training sessions for safety managers will be provided as needed.

In conclusion, this project will generate real-world evidence on the most effective and economically viable adaptive smoking cessation program for implementation in the construction sector. It will inform stakeholders, policymakers, and public health advocates about its potential and lay the groundwork for future studies aimed at reducing smoking and improving the health of construction workers [2].

### Dissemination plans

The University of Miami is committed to enhancing the value of research and advancing public knowledge. We recognize that the public dissemination of our scientific results can facilitate the creation of collaborative efforts. Furthermore, we realize that the proposed project may result in novel data that could benefit the entire research and tobacco control community, which is the target audience for this study. Final research data will be shared openly and in a timely manner while being mindful that the confidentiality and privacy of participants in the proposed research must be protected at all times.

Our research team has devised a two-pronged approach to disseminate and share the research findings and products generated from this research study. Prong #1 includes drafting abstracts, scientific manuscripts, and reports of study findings and lessons learned for broad dissemination in peer-reviewed scientific journals (e.g., Journal of Environmental and Occupational Medicine, American Journal of Public Health, Tobacco Control, Journal of Immigrant and Minority Health). We will also disseminate our research findings at national, state, and local scientific and labor/union conferences. Prong #2 includes the development of fact sheets and policy briefs based on the research findings and lessons learned in developing and evaluating tobacco cessation programs for this high-risk population.

To maximize the impact of the dissemination plan, we have partnered with key stakeholders in industry, union, and government to assist our research team in sharing the study findings with other groups and networks engaged in protecting the health of minority male workers (e.g., Florida Department of Health, Occupational Health and Safety Program, The Center for Construction Research and Training, Occupational Health and Safety Administration Regional Office).

Quality-controlled raw data and processed data used in publications will be made available to allow interested groups to reproduce results from the raw data. All final peer-reviewed manuscripts arising from this proposal will be submitted to the digital archive PubMed Central and made publicly available.

## Supporting information

S1 File The IRB-approved consent form.
(PDF)

S1 Protocol The last IRB-approved version of the study protocol.
(PDF)

## References

[1] Syamlal G, King BA, Mazurek JM. Tobacco product use among workers in the construction industry, United States, 2014‐2016. American Journal of Industrial Medicine. 2018;61(11):939–51. 30229974 10.1002/ajim.22907 PMC6350769

[2] US Department of Health and Human Services, Centers for Disease Control and Prevention, National Center for Chronic Disease Prevention and Health Promotion, Office on Smoking and Health. The Health Consequences of Smoking—50 Years of Progress: A Report of the Surgeon General. 2014.

[3] US Bureau of Labor Statistics. Labor Force Statistics from the Current Population Survey 2021. https://www.bls.gov/cps/cpsaat11.htm

[4] Lee DJ, Fleming LE, Arheart KL, LeBlanc WG, Caban AJ, Chung-Bridges K, et al. Smoking rate trends in U.S. occupational groups: the 1987 to 2004 National Health Interview Survey. J Occup Environ Med. 2007;49(1):75–81. doi: 10.1097/JOM.0b013e31802ec68c 17215716

[5] Lee DJ, Fleming LE, McCollister KE, Caban AJ, Arheart KL, LeBlanc WG, et al. Healthcare provider smoking cessation advice among US worker groups. Tob Control. 2007;16(5):325–8. doi: 10.1136/tc.2006.019117 17897991 PMC2598555

[6] Sorensen G, Barbeau E, Hunt MK, Emmons K. Reducing social disparities in tobacco use: a social-contextual model for reducing tobacco use among blue-collar workers. Am J Public Health. 2004;94(2):230–9. doi: 10.2105/ajph.94.2.230 14759932 PMC1448233

[7] Cahill K, Lancaster T. Workplace interventions for smoking cessation. Cochrane Library. 2014. 10.1002/14651858.CD003440.pub4 PMC11285308 24570145

[8] King BA, Pechacek TF, Mariolis P. Best practices for comprehensive tobacco control programs. U.S. Department of Health and Human Services. 2014.

[9] Syamlal G, King BA, Mazurek JM. Workplace Smoke-Free Policies and Cessation Programs Among U.S. Working Adults. Am J Prev Med. 2019;56(4):548–62. doi: 10.1016/j.amepre.2018.10.030 30772152 PMC6854656

[10] Sorensen G, Barbeau EM, Stoddard AM, Hunt MK, Goldman R, Smith A, et al. Tools for health: the efficacy of a tailored intervention targeted for construction laborers. Cancer Causes Control. 2007;18(1):51–9. doi: 10.1007/s10552-006-0076-9 17186421

[11] Groeneveld IF, Proper KI, van der Beek AJ, Hildebrandt VH, van Mechelen W. Short and long term effects of a lifestyle intervention for construction workers at risk for cardiovascular disease: a randomized controlled trial. BMC Public Health. 2011;11:836. doi: 10.1186/1471-2458-11-836 22040007 PMC3247875

[12] Asfar T, Arheart KL, McClure LA, Ruano-Herreria EC, Dietz NA, Ward KD, et al. Implementing a Novel Workplace Smoking Cessation Intervention Targeting Hispanic/Latino Construction Workers: A Pilot Cluster Randomized Trial. Health Educ Behav. 2021;48(6):795–804. doi: 10.1177/1090198120960395 33063570

[13] Feltner C, Peterson K, Palmieri Weber R, Cluff L, Coker-Schwimmer E, Viswanathan M, et al. The Effectiveness of Total Worker Health Interventions: A Systematic Review for a National Institutes of Health Pathways to Prevention Workshop. Ann Intern Med. 2016;165(4):262–9. doi: 10.7326/M16-0626 27240022

[14] Caban-Martinez A, Asfar T, Ruano-Herreria EC, Arheart KL, McClure LA, Danielle S. Delivering a lunch truck smoking cessation intervention at the construction worksite: perspectives from construction company senior safety leadership. 2019. https://cdn.ymaws.com/www.srnt.org/resource/resmgr/SRNT19_Rapid_Abstracts.pdf

[15] Asfar T, Arheart KL, McClure LA, Ruano-Herreria EC, Dietz NA, Ward KD. Implementing a novel workplace smoking cessation intervention targeting Hispanic/Latino construction workers: A pilot cluster randomized trial. Health Education & Behavior. 2020. 10.1177/1090198120960395 33063570

[16] Asfar T, McClure LA, Arheart KL, Ruano-Herreria EC, Jr Gilford CG, Moore K. Integrating worksite smoking cessation services into the construction sector: opportunities and challenges. Health Education & Behavior. 2019;46(6):1024–34. 31426671 10.1177/1090198119866900

[17] Fiore MC, Jaén CR, Baker TB, Bailey WC, Benowitz NL, Curry SJ. Treating Tobacco Use and Dependence: 2008 Update. Rockville, MD: U.S. Department of Health and Human Services. 2008.

[18] Stead LF, Perera R, Lancaster T. A systematic review of interventions for smokers who contact quitlines. Tob Control. 2007;16 Suppl 1(Suppl 1):i3-8. doi: 10.1136/tc.2006.019737 18048627 PMC2598525

[19] Schauer GL, Malarcher AM, Zhang L, Engstrom MC, Zhu S-H. Prevalence and correlates of quitline awareness and utilization in the United States: an update from the 2009-2010 National Adult Tobacco Survey. Nicotine Tob Res. 2014;16(5):544–53. doi: 10.1093/ntr/ntt181 24253378

[20] McAfee TA. Quitlines a tool for research and dissemination of evidence-based cessation practices. Am J Prev Med. 2007;33(6 Suppl):S357-67. doi: 10.1016/j.amepre.2007.09.011 18021911

[21] Glasgow RE, Klesges LM, Dzewaltowski DA, Bull SS, Estabrooks P. The future of health behavior change research: what is needed to improve translation of research into health promotion practice?. Ann Behav Med. 2004;27(1):3–12. doi: 10.1207/s15324796abm2701_2 14979858

[22] Andrews JO, Felton G, Ellen Wewers M, Waller J, Tingen M. The effect of a multi-component smoking cessation intervention in African American women residing in public housing. Res Nurs Health. 2007;30(1):45–60. doi: 10.1002/nur.20174 17243107

[23] Curry SJ, Ludman EJ, Graham E, Stout J, Grothaus L, Lozano P. Pediatric-based smoking cessation intervention for low-income women: a randomized trial. Arch Pediatr Adolesc Med. 2003;157(3):295–302. 12622686 10.1001/archpedi.157.3.295

[24] Clauser SB, Taplin SH, Foster MK, Fagan P, Kaluzny AD. Multilevel intervention research: lessons learned and pathways forward. J Natl Cancer Inst Monogr. 2012;2012(44):127–33. doi: 10.1093/jncimonographs/lgs019 22623606 PMC3482966

[25] Yano EM, Green LW, Glanz K, Ayanian JZ, Mittman BS, Chollette V, et al. Implementation and spread of interventions into the multilevel context of routine practice and policy: implications for the cancer care continuum. J Natl Cancer Inst Monogr. 2012;2012(44):86–99. doi: 10.1093/jncimonographs/lgs004 22623601 PMC3482959

[26] Charns MP, Foster MK, Alligood EC, Benzer JK, Jr Burgess JF, Li D, et al. Multilevel interventions: measurement and measures. J Natl Cancer Inst Monogr. 2012;2012(44):67–77. doi: 10.1093/jncimonographs/lgs011 22623598 PMC3482970

[27] Collins LM, Murphy SA, Bierman KL. A conceptual framework for adaptive preventive interventions. Prev Sci. 2004;5(3):185–96. doi: 10.1023/b:prev.0000037641.26017.00 15470938 PMC3544191

[28] Dawson R, Lavori PW. Efficient design and inference for multistage randomized trials of individualized treatment policies. Biostatistics. 2012;13(1):142–52. doi: 10.1093/biostatistics/kxr016 21765180 PMC3276275

[29] Murphy SA. An experimental design for the development of adaptive treatment strategies. Stat Med. 2005;24(10):1455–81. doi: 10.1002/sim.2022 15586395

[30] Curran GM, Bauer M, Mittman B, Pyne JM, Stetler C. Effectiveness-implementation hybrid designs: combining elements of clinical effectiveness and implementation research to enhance public health impact. Med Care. 2012;50(3):217–26. doi: 10.1097/MLR.0b013e3182408812 22310560 PMC3731143

[31] Powell BJ, Waltz TJ, Chinman MJ, Damschroder LJ, Smith JL, Matthieu MM, et al. A refined compilation of implementation strategies: results from the Expert Recommendations for Implementing Change (ERIC) project. Implement Sci. 2015;10:21. doi: 10.1186/s13012-015-0209-1 25889199 PMC4328074

[32] Glasgow RE, McKay HG, Piette JD, Reynolds KD. The RE-AIM framework for evaluating interventions: what can it tell us about approaches to chronic illness management?. Patient Educ Couns. 2001;44(2):119–27. doi: 10.1016/s0738-3991(00)00186-5 11479052

[33] Glasgow RE, Emmons KM. How can we increase translation of research into practice? Types of evidence needed. Annu Rev Public Health. 2007;28:413–33. doi: 10.1146/annurev.publhealth.28.021406.144145 17150029

[34] Glasgow RE, Vogt TM, Boles SM. Evaluating the public health impact of health promotion interventions: the RE-AIM framework. Am J Public Health. 1999;89(9):1322–7. doi: 10.2105/ajph.89.9.1322 10474547 PMC1508772

[35] Glasgow RE, Harden SM, Gaglio B, Rabin B, Smith ML, Porter GC, et al. RE-AIM Planning and Evaluation Framework: Adapting to New Science and Practice With a 20-Year Review. Front Public Health. 2019;7:64. doi: 10.3389/fpubh.2019.00064 30984733 PMC6450067

[36] Carroll C, Patterson M, Wood S, Booth A, Rick J, Balain S. A conceptual framework for implementation fidelity. Implement Sci. 2007;2:40. doi: 10.1186/1748-5908-2-40 18053122 PMC2213686

[37] Landes SJ, McBain SA, Curran GM. Reprint of: An introduction to effectiveness-implementation hybrid designs. Psychiatry Res. 2020;283:112630. doi: 10.1016/j.psychres.2019.112630 31722790

[38] Dietz NA, Asfar T, Caban-Martinez AJ, Ward KD, Santiago K, Ruano-Herreria EC, et al. Developing a Worksite-based Culturally Adapted Smoking Cessation Intervention for Male Hispanic/Latino Construction Workers. J Smok Cessat. 2019;14(2):73–82. doi: 10.1017/jsc.2018.16 31073339 PMC6502474

[39] Hartmann‐Boyce J, Hong B, Livingstone‐Banks J, Wheat H, Fanshawe TR. Additional behavioural support as an adjunct to pharmacotherapy for smoking cessation. Cochrane Database of Systematic Reviews. 2019;(6). 10.1002/14651858.CD009670.pub4 PMC6549450 31166007

[40] Stead LF, Lancaster T. Behavioural interventions as adjuncts to pharmacotherapy for smoking cessation. Cochrane Database Syst Rev. 2012;12:CD009670. doi: 10.1002/14651858.CD009670.pub2 23235680

[41] Stead LF, Koilpillai P, Lancaster T. Additional behavioural support as an adjunct to pharmacotherapy for smoking cessation. Cochrane Database of Systematic Reviews. 2015;2015(10). 10.1002/14651858.CD009670.pub3 26457723

[42] Bandura A. Health promotion from the perspective of social cognitive theory. Psychology & Health. 1998;13(4):623–49. doi: 10.1080/08870449808407422

[43] Bandura A. Social learning theory. Englewood Cliffs, N.J: Prentice-Hall. 1977.

[44] Dietz NA, Asfar T, Caban-Martinez AJ, Ward KD, Santiago K, Ruano-Herreria EC. Developing a worksite-based culturally adapted smoking cessation intervention for male Hispanic/Latino construction workers. Journal of Smoking Cessation. 2018;:1–10. 31073339 10.1017/jsc.2018.16 PMC6502474

[45] Asfar T, Caban-Martinez AJ, McClure LA, Ruano-Herreria EC, Sierra D, Jr Gilford Clark G, et al. A cluster randomized pilot trial of a tailored worksite smoking cessation intervention targeting Hispanic/Latino construction workers: Intervention development and research design. Contemp Clin Trials. 2018;67:47–55. doi: 10.1016/j.cct.2018.02.007 29454141 PMC6377564

[46] Lancaster T, Stead L. Individual behavioural counselling for smoking cessation. Cochrane Database Syst Rev. 2005;2. 10.1002/14651858.CD001292.pub2 15846616

[47] Lancaster T, Stead LF. Self-help interventions for smoking cessation. Cochrane Database Syst Rev. 2005;3(3). 10.1002/14651858.CD001118.pub2 16034855

[48] Stead LF, Perera R, Lancaster T. Telephone counselling for smoking cessation. Cochrane Database Syst Rev. 2006;3. 10.1002/14651858.CD002850.pub2 16855992

[49] Piper ME, McCarthy DE, Baker TB. Assessing tobacco dependence: a guide to measure evaluation and selection. Nicotine Tob Res. 2006;8(3):339–51. doi: 10.1080/14622200600672765 16801292

[50] Vilela FADB, Jungerman FS, Laranjeira R, Callaghan R. The transtheoretical model and substance dependence: theoretical and practical aspects. Braz J Psychiatry. 2009;31(4):362–8. doi: 10.1590/s1516-44462009005000010 19918675

[51] Biener L, Abrams DB. The Contemplation Ladder: validation of a measure of readiness to consider smoking cessation. Health Psychol. 1991;10(5):360–5. doi: 10.1037//0278-6133.10.5.360 1935872

[52] Hendricks PS, Wood SB, Baker MR, Delucchi KL, Hall SM. The Smoking Abstinence Questionnaire: measurement of smokers’ abstinence-related expectancies. Addiction. 2011;106(4):716–28. doi: 10.1111/j.1360-0443.2010.03338.x 21205053 PMC3348861

[53] Reuland DS, Cherrington A, Watkins GS, Bradford DW, Blanco RA, Gaynes BN. Diagnostic accuracy of Spanish language depression-screening instruments. Ann Fam Med. 2009;7(5):455–62. doi: 10.1370/afm.981 19752474 PMC2746515

[54] Shea T, De Cieri H. Workplace stress evaluation tools: A Snapshot Review. 2011.

[55] Humeniuk R, Ali R, Babor TF, Farrell M, Formigoni ML, Jittiwutikarn J, et al. Validation of the Alcohol, Smoking And Substance Involvement Screening Test (ASSIST). Addiction. 2008;103(6):1039–47. doi: 10.1111/j.1360-0443.2007.02114.x 18373724

[56] Zimet GD, Dahlem NW, Zimet SG, Farley GK. The Multidimensional Scale of Perceived Social Support. Journal of Personality Assessment. 1988;52(1):30–41. doi: 10.1207/s15327752jpa5201_2 2280326

[57] World Health Organization. Global Tobacco Surveillance System (GTSS). Tobacco Questions for Surveys 2011. 2011.

[58] Brown RA, Burgess ES, Sales SD, Whiteley JA, Evans DM, Miller IW. Reliability and validity of a smoking timeline follow-back interview. Psychology of Addictive Behaviors. 1998;12(2):101–12. doi: 10.1037/0893-164x.12.2.101

[59] Hughes JR, Hatsukami D. Signs and symptoms of tobacco withdrawal. Arch Gen Psychiatry. 1986;43(3):289–94. doi: 10.1001/archpsyc.1986.01800030107013 3954551

[60] Cox LS, Tiffany ST, Christen AG. Evaluation of the brief questionnaire of smoking urges (QSU-brief) in laboratory and clinical settings. Nicotine Tob Res. 2001;3(1):7–16. doi: 10.1080/14622200020032051 11260806

[61] Benowitz NL, Jacob P, Hall S, Tsoh J, Ahijevych K, Jarvis M, et al. Biochemical verification of tobacco use and cessation. Nicotine Tob Res. 2002;4(2):149–59. doi: 10.1080/14622200210123581 12028847

[62] Piper ME, Bullen C, Krishnan-Sarin S, Rigotti NA, Steinberg ML, Streck JM, et al. Defining and Measuring Abstinence in Clinical Trials of Smoking Cessation Interventions: An Updated Review. Nicotine Tob Res. 2020;22(7):1098–106. doi: 10.1093/ntr/ntz110 31271211 PMC9633719

[63] French MT. Drug abuse treatment cost analysis program (DATCAP): Program version user’s manual. Eighth Edition ed. University of Miami. 2001.

[64] Helfrich CD, Li Y-F, Sharp ND, Sales AE. Organizational readiness to change assessment (ORCA): development of an instrument based on the Promoting Action on Research in Health Services (PARIHS) framework. Implement Sci. 2009;4:38. doi: 10.1186/1748-5908-4-38 19594942 PMC2716295

[65] Proctor E, Silmere H, Raghavan R, Hovmand P, Aarons G, Bunger A, et al. Outcomes for implementation research: conceptual distinctions, measurement challenges, and research agenda. Adm Policy Ment Health. 2011;38(2):65–76. doi: 10.1007/s10488-010-0319-7 20957426 PMC3068522

[66] Attkisson CC, Greenfield TK. Client Satisfaction Questionnaire-8 and Service Satisfaction Scale-30. 1994.

[67] Luke DA, Calhoun A, Robichaux CB, Elliott MB, Moreland-Russell S. The Program Sustainability Assessment Tool: a new instrument for public health programs. Prev Chronic Dis. 2014;11:130184. doi: 10.5888/pcd11.130184 24456645 PMC3900326

[68] Asfar T, Klesges RC, Sanford SD, Sherrill-Mittleman D, Robison LL, Hudson MM, et al. Trial design: The St. Jude Children’s Research Hospital Cancer Survivors Tobacco Quit Line study. Contemp Clin Trials. 2010;31(1):82–91. doi: 10.1016/j.cct.2009.09.004 19766734 PMC2818168

[69] Asfar T, Weg MV, Maziak W, Hammal F, Eissenberg T, Ward KD. Outcomes and adherence in Syria’s first smoking cessation trial. Am J Health Behav. 2008;32(2):146–56. doi: 10.5555/ajhb.2008.32.2.146 18052855

[70] Ward KD, Asfar T, Al Ali R, Rastam S, Vander Weg MW, Eissenberg T. Randomized trial of the effectiveness of combined behavioral/pharmacological smoking cessation treatment in Syrian primary care clinics. Addiction. 2012;n/a:n/a. 10.1111/j.1360-0443.2012.04048.x PMC7942391 22882805

[71] Chakraborty B, Murphy SA. Dynamic Treatment Regimes. Annu Rev Stat Appl. 2014;1:447–64. doi: 10.1146/annurev-statistics-022513-115553 25401119 PMC4231831

[72] Lavori PW, Dawson R. Introduction to dynamic treatment strategies and sequential multiple assignment randomization. Clin Trials. 2014;11(4):393–9. doi: 10.1177/1740774514527651 24784487 PMC4216645

[73] Murray DM. Design and analysis of group-randomized trials. USA: Oxford University Press. 1998.

[74] Zeger SL, Liang KY. Longitudinal data analysis for discrete and continuous outcomes. Biometrics. 1986;42(1):121–30. 3719049

[75] Wang M, Kong L, Li Z, Zhang L. Covariance estimators for generalized estimating equations (GEE) in longitudinal analysis with small samples. Stat Med. 2016;35(10):1706–21. doi: 10.1002/sim.6817 26585756 PMC4826860

[76] Nahum-Shani I, Almirall D, Yap JRT, McKay JR, Lynch KG, Freiheit EA, et al. SMART longitudinal analysis: A tutorial for using repeated outcome measures from SMART studies to compare adaptive interventions. Psychol Methods. 2020;25(1):1–29. doi: 10.1037/met0000219 31318231 PMC7480232

[77] Donner A. Some Aspects of the Design and Analysis of Cluster Randomization Trials. Journal of the Royal Statistical Society Series C: Applied Statistics. 1998;47(1):95–113. doi: 10.1111/1467-9876.00100

[78] Bender R, Lange S. Adjusting for multiple testing--when and how?. J Clin Epidemiol. 2001;54(4):343–9. doi: 10.1016/s0895-4356(00)00314-0 11297884

[79] Sanders GD, Neumann PJ, Basu A, Brock DW, Feeny D, Krahn M, et al. Recommendations for Conduct, Methodological Practices, and Reporting of Cost-effectiveness Analyses: Second Panel on Cost-Effectiveness in Health and Medicine. JAMA. 2016;316(10):1093–103. doi: 10.1001/jama.2016.12195 27623463

[80] Group TE. EuroQol--a new facility for the measurement of health-related quality of life. Health Policy. 1990;16(3):199–208. doi: 10.1016/0168-8510(90)90421-9 10109801

[81] Loomes G, McKenzie L. The use of QALYs in health care decision making. Soc Sci Med. 1989;28(4):299–308. doi: 10.1016/0277-9536(89)90030-0 2649989

[82] Baltussen RM, Hutubessy RC, Evans DB, Murray CJ. Uncertainty in cost-effectiveness analysis. Probabilistic uncertainty analysis and stochastic league tables. Int J Technol Assess Health Care. 2002;18(1):112–9. 11987434

[83] Briggs AH, Mooney CZ, Wonderling DE. Constructing confidence intervals for cost-effectiveness ratios: an evaluation of parametric and non-parametric techniques using Monte Carlo simulation. Stat Med. 1999;18(23):3245–62. doi: 10.1002/(sici)1097-0258(19991215)18:23<3245::aid-sim314>3.0.co;2-2 10602149

[84] Shaw JW, Johnson JA, Coons SJ. US valuation of the EQ-5D health states: development and testing of the D1 valuation model. Med Care. 2005;43(3):203–20. doi: 10.1097/00005650-200503000-00003 15725977

[85] CONSORT. Consolidated Standards of Reporting Trials. 2010 http://www.consort-statement.org/10.1038/sj.bdj.4800065a 10205958

[86] Thorpe KE, Zwarenstein M, Oxman AD, Treweek S, Furberg CD, Altman DG, et al. A pragmatic-explanatory continuum indicator summary (PRECIS): a tool to help trial designers. J Clin Epidemiol. 2009;62(5):464–75. doi: 10.1016/j.jclinepi.2008.12.011 19348971

[87] Census Bur. Statistical Abstract of the United States: 2000. 120th Edition. 2000. https://www.census.gov/library/publications/2000/compendia/statab/120ed.html

[88] Tunis SR, Stryer DB, Clancy CM. Practical clinical trials: increasing the value of clinical research for decision making in clinical and health policy. JAMA. 2003;290(12):1624–32. doi: 10.1001/jama.290.12.1624 14506122

[89] Zwarenstein M, Treweek S, Gagnier JJ, Altman DG, Tunis S, Haynes B, et al. Improving the reporting of pragmatic trials: an extension of the CONSORT statement. BMJ. 2008;337:a2390. doi: 10.1136/bmj.a2390 19001484 PMC3266844

