# Supplementary material for: Empowering safety managers to champion the implementation of smoking cessation services in the construction industry: Protocol for a sequential multiple assignment randomized trial
Source: PLoS One. 2025 Jun 9;20(6):e0324717. doi: 10.1371/journal.pone.0324717 (PMC12148188; doi:10.1371/journal.pone.0324717)
Supplement: S1 Protocol — The last IRB-approved version of the study protocol. (PDF) [file pone.0324717.s002.pdf]

Version 6
December 02, 2024

**Principal Investigator**	Taghrid Asfar, MD, MSPH<br>University of Miami Department of Public Health Sciences<br>1120 NW 14 St<br>Miami, FL 33136<br>(305) 243-3826
**IRB Number (eProst ID):**	20230549
**Protocol Title:**	Expanding the role of the safety manager to implement a workplace smoking cessation program in the construction sector.
**Funding Agency**	Florida Department of Health<br>James and Esther King
**NCT #**	NCT06098144
**Version #**	6
**Version Date:**	December 02, 2024

1) Protocol Title

Expanding the role of the safety manager to implement a workplace smoking cessation program in the construction sector.

2) Objective*

The main objective of this study is to identify the optimal adaptive smoking cessation program for the construction sector in terms of effectiveness, cost-effectiveness, and potential implementation.

3) Background*

Tobacco use among US construction workers (CWs) is a major health disparity affecting minority groups. The smoking rate among CWs is double that of the general population, and almost half of CWs are racial/ethnic minorities, with low income and limited access to smoking cessation services. While implementing workplace smoking cessation programs among CWs has been recognized as a top priority, no systematic development and implementation of such programs have been attempted. Only 3 prior trials tested smoking cessation programs among US CWs, and all were single-level studies exclusively focused on individuals (CWs), without consideration of factors that might influence program adoption at the organizational level. In addition, an important gap in current knowledge is the lack of evidence about the optimal intensity for effective/cost-effective smoking cessation programs to be implemented in the construction sector. Supported by an NIH/NCI grant (R21CA202993), we recently developed and pilot-tested a highly scalable workplace smoking cessation program that addresses the organizational structure, work environment, and CWs' individual factors. For further development and implementation, leaders in the construction sector recommended testing 3 programs with increasing intensity to identify the optimal adaptive program for implementation in the construction sector: A) Tobacco Quitline (TQL); B) (our brief program) TQL + nicotine replacement therapy (NRT) + 1 group behavioral counseling session; and C) (high-intensity program) NRT + 4 group behavioral counseling sessions; with all 3 delivered onsite by the safety manager. In response, we propose to test the 3 programs proposed by company leaders to identify the optimal adaptive program in terms of effectiveness, cost-effectiveness, and potential implementation. Findings will inform practice and policy to eliminate tobacco-related health disparities among CWs by implementing sustainable smoking cessation services in the construction sector.

In summary, this study is significant because it will: 1) focus on CWs who are disproportionately impacted by tobacco-related illnesses; 2) address a critical scientific gap in research on workplace smoking cessation programs among CWs who are traditionally under-represented; 3) involve collaboration with leaders and safety managers in the construction sector to ensure impact, feasibility and sustainability of the

proposed work; 4) involve the development of MLIs tailored to the construction sector organizational structure and CWs; and 5) generate practical, real-world evidence on the optimal adaptive smoking cessation program in terms of effectiveness, cost-effectiveness, and potential implementation in the construction.

4) **Inclusion***

Company Leaders who are eligible for inclusion are those who are:
1) ≥18 years old
2) Involved in decision-making
3) Willing to participate in the implementation development and evaluation

Safety managers who are eligible for inclusion are those who are:
1) Self-identified as a safety manager or superintendent
2) Have no plans to leave the company in the next year
3) Speak either English or Spanish
4) Non-cigarette smoker
5) Willing to receive training in smoking cessation treatment
6) Willing to deliver the smoking cessation treatment to workers

The inclusion criteria for CWs in the intervention are:
1) ≥18 years old
2) Being a construction worker in the participating construction companies
3) Have smoked an average of ≥5 cigarettes/day in the past year (those who use other tobacco products such as smokeless tobacco or e-cigarettes will be included in the study for generalizability. However, their use will be monitored during the study, and participants will be encouraged to quit all tobacco methods).
4) Willing to make a serious quit attempt in the next 30 days
5) Willing to receive treatment from the Florida Tobacco Quitline
6) Have no plans to leave the company in the next 6 months
7) Own a telephone and plan to keep it active for the next 12 months

The inclusion criteria for CWs participating in the *tobacco use pre-implementation assessment* are:
1) Age ≥18 years old
2) Being a construction worker in the participating construction companies

5) **Procedures Involved***

Study 1. Developing and testing the program implementation strategies

We will (1) develop multilevel implementation strategies (MLIs) for workplace smoking cessation programs delivered by the safety managers, and (2) conduct three pre-, mid-, and post-implementation process evaluations to examine the feasibility of the program implementation.

(1) develop MLIs

Keeping in mind that our implementation strategies will not be finalized until completing the pre-implementation assessment, we present below tentative implementation strategies based on our consultation with leaders and safety managers who agreed to participate in the study:

a. Build a coalition to develop an inner support system (internal facilitation): An "Advisory Committee" will be formed by each construction company to guide the development and documentation of the program implementation within their work environment. Each committee will include 1) a study "champion" who will play a liaison role between the company and the research staff and facilitate and oversee all on-site implementation activities, 2) a representative for safety managers who will deliver the program, and 3) a representative for CWs.

b. Provide external facilitation: The study PI (Asfar), two Co-Is (Lee and Salloum), two Research Assistants (RAs), and one graduate student with backgrounds in public health will: 1) provide ongoing consultation on program training and delivery, help problem solve, and offer ongoing technical support for developing and implementing tools for data collection, quality monitoring, and training throughout the program implementation process; 2) track all facilitation activities, report on implementation status and progress, and complete the implementation evaluation; and 3) have a bimonthly videoconference with the company's advisory committees throughout the project to discuss progress and improvement in implementation and give input on study procedures, outcome assessments, and study dissemination.

c. Assess for readiness and identify barriers and facilitators: The research team will conduct a pre-implementation assessment meeting with opinion leaders, safety managers, and representatives of CWs to present the scientific evidence behind the program and its benefits to the organization. The meeting will also include a discussion on how the program should best be implemented to fit the organizational context and work environment and the expected barriers and facilitators.

d. Perform an observational field assessment of workflow on construction sites: We will shadow safety managers and conduct informal interviews to identify implementation barriers and facilitators. The assessment will provide perspective on the needs and assets of the existing workflow to minimize disruption and enhance program delivery and integrity.

e. Develop an implementation activities checklist: We will create an implementation activities checklist based on our pre-assessment that will guide the implementation

process. We will meet with each company's Advisory Committee to review and revise the activities checklist based on their local needs. We will attempt to adapt the planned activities for each company while retaining the core elements.

f. Training safety managers to deliver the program: Safety managers who agree to participate in the study will undergo one day of training by Asfar in: 1) study protocol, 2) human subjects protection, 3) clinical documentation using REDCap (a cloud-based web survey tool provided for free by UM) downloaded on iPads to report and track their activities during the program implementation, 4) program fidelity, and 5) practical experience in program delivery through a series of standardized participant encounters that involve providing the program via study protocol to an actor trained to portray a worker enrolled in the study. All encounters will be audiotaped and reviewed by Asfar and Lee to determine if safety managers have met the training objectives. Safety managers also will complete a 10-hour self-paced tobacco treatment online via the Florida Area Health Education Centers Network (AHEC) Cessation Program. This online program provides online tobacco cessation educational modules to deliver effective evidence-based tobacco use treatment and cessation services.

g. Develop and organize quality monitoring system: To ensure the quality of execution, we will track and document all implementation activities in a time motion tracking log to assess the fidelity and intensity (quality and depth) of implementation [119], timeliness of task completion, and degree of engagement of key involved individuals (e.g., leaders, champion, safety managers) throughout the process.

<u>(2) conduct three pre-, mid-, and post-implementation process evaluations:</u>

We will conduct three mixed-methods quantitative and qualitative process evaluations (pre-, mid-, post-implementation) to help improve the program implementation strategies, formulate interpretations of primary outcomes data (e.g., cessation rate), and create recommendations for future implementation. Each evaluation will be conducted among company leaders (n = 12-18), safety managers (n = 20-25), and CWs (n = 36, from the SMART study), and will include 1) key-informant interviews (safety managers, leaders), 2) surveys (leaders, safety managers, workers), and 3) a qualitative assessment of documented notes and discussions from the bimonthly meetings of the investigators and advisory committee. In addition, we will conduct a baseline assessment of tobacco use among all workers in the participating companies to be able to examine the program reach (reach = the number of all smokers/number of smokers enrolled in the study).

a. <u>In-depth key-informant interviews:</u> As part of the implementation process evaluation, we will conduct semi-structured interviews that last ≈ 20-30 minutes 3 times (pre-implementation, mid-implementation, and post-implementation) with company leaders (n = 12-18), safety manager (n = 20-25). Informed by CFIR, the interview guide will focus on perceptions regarding evidence, inner/outer context, and barriers and facilitators of implementation, including: 1) a short series of closed-ended questions (e.g., demographics, job-characteristics), and 2) open-ended questions to explore

perceptions of program marketing, sustainability, barriers and facilitators to implementation, suggestions for improvement, and goals and expectations for the current study. Interviews will be audio-recorded and professionally transcribed for analysis. Data will be entered in NVivo version 12. Data analysis will include a hybrid inductive-deductive approach, with deductive codes informed by the CFIR's five domains and inductive codes derived from emerging themes. Collaborative coding, data reduction, display, and interpretation will be conducted by Asfar in consultation with Dr. Salloum using Creswell's 7-step analysis framework. Overlapping themes will be given greater emphasis. All initial codes and categories will be sorted and compared until core categories of recurrent themes emerge. Findings will be presented to the Advisory Committee during meetings to inform implementation efforts. A similar analysis will be conducted on the documented notes and discussions from these meetings to assess the advisory committee's performance during the study.

b. <u>Surveys:</u> We will conduct surveys administered through REDCap among leaders (n ≈ 12 - 18), safety managers (n ≈ 20 - 25), and CWs (n ≈ 36; randomly selected from SMART). Assessments will measure: 1) evidence supporting the program; 2) implementation feasibility (utility/suitability for everyday use, TQL enrollment rates); [3) implementation acceptability including CWs satisfaction with the program (using the Client Satisfaction Questionnaire), safety managers' training and incentives, comfort, and delivery; 4) barriers to program implementation; 5) changes in implementation self-efficacy and behavior capabilities; and 6) the Organizational Readiness to Change Assessment (ORCA). To assess program adoption, we will conduct a monthly assessment of 1) the proportion of CWs who are screened, enrolled, and engaged in the treatment, and 2) the proportion of safety managers who are providing the treatment. We will assess the programs' short-term (3-month) sustainability at the individual and setting levels. At the individual level, we will examine monthly enrollment rates to examine time trends in enrollment and whether CWs and safety managers continue to use the program during the 3 months after completing the study. At the setting level, we will use the Intervention Sustainability Assessment Tool (PSAT) to assess each program's current capacity for sustainability across a range of specific organizational and contextual factors (e.g., capacity, environmental support). Numbers (%) will be used to present data from categorical variables in the surveys. Continuous data will be presented as means [+/-SD].

   In addition, we will conduct a tobacco use assessment among all workers in the participating companies to be able to examine the program's reach. To do so, we added a new consent form and a brief survey instrument for the assessment.

Study 2. Cluster, hybrid type 1 effectiveness-implementation, 2-phase SMART Clinical Trial

We will conduct a pragmatic, 2-arm, cluster, hybrid type 1 effectiveness-implementation, 2-phase SMART to test the 3 programs proposed by leaders: A) TQL; B) TQL + NRT + 1 brief group behavioral counseling session; and C) NRT + 4 intensive group behavioral counseling sessions. We removed the NRT from group A because the Florida TQLs provide up to 12 weeks of NRT.

We will recruit 32 construction sites within 8 construction companies with 608 CWs who are smokers. In Phase 1, construction sites will be randomly assigned to two conditions in a 1:1 ratio (A or B; 16 sites/group). Study participants (n=608; 304/arm) working in these companies will receive treatment delivered by the safety managers following the regular daily safety briefings. In Phase 2, responders (CWs who quit smoking at the 3-month assessment based on self-reported abstinence (defined as not smoking even, a puff, after 15 days of quitting date until now)validated by saliva cotinine tests) will continue in the same treatment (conditions A1 & B1). Non-responders (did not quit smoking at 3-month assessment) will be re-randomized either to C (conditions A1+C & B1+C) or to the same treatment (conditions A2 & B2) in a 1:1 ratio adjusted depending on the rate of non-response in Phase 1. Randomization in Phase 1 will be stratified by company instead of sites, to avoid cross over contamination by workers who may be working at multiple sites simultaneously. Worker-level randomization in Phase 2 will be stratified by company and by the treatment groups in Phase 1 of SMART. In Phase 1, participants in A1 and B1 will receive a 3-month follow-up to determine they quit smoking. In Phase 2, all participants will receive 3 follow-up assessments at 6, 9, and 12 months after enrollment. These follow-up assessments will assess smoking status (using the timeline follow-back measure [145]), use of additional NRT or cessation drugs (e.g., bupropion), use of other tobacco products, and number/time of contact with the safety manager and TQL. Biochemical validation of smoking status via salivary sample for cotinine analysis will be obtained from those who self-report quitting cigarettes (not smoking, even a puff, after 15 days of quit date until now) at the 3-month and 12-month assessment. Saliva samples will be collected using the NicoTests® Cotinine Test Device for the detection of Cotinine, a metabolite of nicotine. This test detects Cotinine in oral fluid/saliva at cut-off concentrations of 30 ng/mL, the standard US level for oral fluid nicotine/cotinine tests. Saliva concentrations of Cotinine reflect blood levels.

In the SMART study, participating CWs will receive a total of $100 incentives ($20x5 assessments). Safety managers will receive a $500 gift card for participating in the study.

In the implementation process evaluation, CWs who complete the tobacco use assessment survey will receive $10. In addition, CWs ($n \approx 36$; randomly selected from SMART) and safety managers ($n \approx 20$) will receive an additional $120 for completing 3 surveys ($20 each) and 3 interviews ($20 each). Participating leaders ($n \approx 12$) in the key informant interviews and the surveys will receive UM memorabilia (t-shirt, cup, bag).

Nicotine Replacement Treatment

Given the highly active nature of CWs job, excessive sweating might affect the stability of the nicotine patches. Therefore, we will use the Nicorette™ gums (2 mg, 4

mg) to help stop smoking. We will determine the initial dose based on self-reported cigarette consumption (see Table 1). NRT will be distributed in Phases 1 and 2 based on the treatment condition. In Phase 1, participants in Group B will receive 3 weeks of NRT treatment in session 1 after the referral to the FL TQLs, and another 3 weeks at the second follow-up (2 weeks after the quit date). In Phase 2, participants in B2 will receive 3 weeks of NRT treatment in Session 1 and another 3 weeks at the second follow-up (2 weeks after the quit date). Participants in Group C will receive 3 weeks of treatment in Session 1 and another 3 weeks of treatment in Session 4. All participants will receive detailed instructions regarding proper gum use and side effects. We will monitor concomitant smoking during NRT use and the use of additional NRT or other cessation drugs (e.g., bupropion).

Table 1. Nicotine gum dose based on the number of cigarettes smoked per day

Number cigarettes smoking	Starting dose of Nicotine Gum	Dosing Schedule	Maximum pieces per day
Greater than 20 cigarettes/day	4mg	4 mg every 1-2 hours	Max 8 pieces a day
Less than 20 cigarettes/day	2mg	2 mg every 1-2 hours	Max 8 pieces a day

Nicotine Gum Use.

- Never smoke and use nicotine gum at the same time
- Use gum only when you have a craving for a cigarette
- Do NOT treat it like chewing gum. It is not to be constantly chewed
- Chew approximately 10-15 times until you get a peppery or tingling feel in your mouth, and then “park” the gum between the cheek and gum on one side of the mouth or the other
- Once the tingling/peppery taste disappears, chew another 10-15 times until the sensation returns, and then park the gum on the other side of the mouth
- Repeat for up to 30 minutes
- It may be irritating to the inside of your mouth, so move it from spot to spot when you park it; and
- Avoid acidic beverages (e.g., coffee, juice, soft drinks) for at least 15 minutes prior to using gum.

Nicotine Gum Side Effects.

Although most persons do not experience any major side effects from nicotine gum, it is possible that a participant could experience any of the following side effects. All participants will be questioned or evaluated for these specific side effects at each session after the gum is dispensed.

- Mouth soreness. Very rare. The probability of occurrence is low, and the risk is low.

- Hiccups. This typically occurs through improper use of the gum. If participants report this, we work on how they are using the gum. Probability is common in those who misuse the gum; risk is low
- Dyspepsia. The probability and risk are low.
- Jaw ache. The probability and risk are low.

Management of Adverse Events Potentially Related to the Study Medication.
If you are experiencing any major side effects, please call us at this phone number: (305) 243-0362 or (786) 505-2920.

Study's Assessments:

1. Baseline Assessment. Eligible CWs participants will undergo a 15-20 min *Baseline Assessment* after consent is given. The baseline assessment will collect information about demographics (age, race/ethnicity, sex, education, marital status), job characteristics, work-related injuries, acculturation, the General Ethnic Discrimination Scale, job stress, ASSIST for alcohol and substance use assessment, social support, and quality-of-life (EQ-5D-3L). Tobacco smoking history and dependence will be assessed using the Fagerström Test for Nicotine Dependence, with additional items assessing the use of e-cigarettes and other tobacco products, exposure to secondhand smoke, salivary cotinine and exhaled carbon monoxide. Depressive symptom assessments include the Perceived Stress Scale and Center for Epidemiological Studies Depression Scale-10 (CES-D). Saliva samples will be collected using the NicoTests®.This test detects Cotinine in oral fluid/saliva at cut-off concentrations of 30 ng/mL (< 30 indicates abstinence). Carbon monoxide breath test results will be collected using a CO Check Pro carbon monoxide breath monitor from Medical Device Depot, with an expired carbon monoxide (CO) level cutoff of <6 ppm.

2. Three-month assessment (Group A1, B1). At the end of Phase 1, all participants in Groups A1 and B1 will receive a 3-month assessment to collect information about their enrollment in the FL TQL, the number/time of total contact with TQL, and their smoking status to determine if they quit smoking. Biochemical validation of smoking status via salivary sample for cotinine analysis will be obtained from those who self-report quitting cigarettes. Saliva samples will be collected using the NicoTests®.This test detects Cotinine in oral fluid/saliva at cut-off concentrations of 30 ng/mL (< 30 indicates abstinence).

3. Intra-treatment (Group C). In Phase 2, participants in Group C will complete an assessment at sessions 3 and 4, including depressive symptoms, the Minnesota Withdrawal Scale, and the Questionnaire of Smoking Urges-Brief Scale. Participants will also record the number of cigarettes/day and nicotine Gums used since the previous session using timeline follow-back calendars.

4. End-of-treatment (Group C). In Phase 2, at 8 weeks after the quit date, participants in Group C will complete an end-of-treatment assessment, including smoking status, the number/time of total contact with the safety manager, perceived stress, depressive symptoms, nicotine withdrawal, other tobacco use products, and the Group Climate Questionnaire. Biochemical validation of smoking status via salivary sample collected using the NicoTests®.This test detects Cotinine in oral fluid/saliva at cut-off concentrations of 30 ng/mL (< 30 indicates abstinence)

5. Six-, nine-, and twelve-month assessment (All Groups). All participants (those who quit smoking in Phase 1, and those who were included in Phase 2) will receive 3 follow-ups at 6-, 9-, and 12-months after enrollment to assess smoking status (using the time-line follow-back measure), concomitant smoking and NRT use, use of other cessation drugs, use of other tobacco products (e.g., e-cigarettes), number/time of total contact with interventionist and TQL, depressive symptoms, the Minnesota Withdrawal Scale, and the Smoking Urges-Brief Scale. The smoking status of those who reported quitting smoking at 12 months will be verified using the NicoTests®. This test detects cotinine in oral fluid/saliva at cut-off concentrations of 30 ng/mL (< 30 indicates abstinence). This will be done in-person or remotely via a HIPAA authorized Zoom meeting.the

<u>Intervention:</u>

This study will test 3 programs:

<u>Group A1 and A2:</u> CWs in this group will be referred to the TQL by the research team using the "Automated online referral form." Participants will be informed that the TQL is free, and the TQL counselor will provide three phone counseling sessions to devise a specific plan to quit smoking and arrange the delivery of 4 to 12 weeks of NRT based on need. Participants will be advised to request nicotine gums instead of patches to accommodate their job circumstances (excessive heat).

<u>Group B1 and B2:</u> In addition to A1 and A2, CWs in B1 and B2 will receive 6 weeks of NRT, a brief (15-20 min) in-person behavioral group counseling session and two brief follow-ups (in-person or by phone) (Table 4). The behavioral counseling session has been developed based on the SCT, our formative data, and cessation literature. The session will discuss preparing to quit, coping with job-related stress, getting social support, the "5 A's" for preventing relapse (Avoid, Alter, Alternatives, Anticipate, and Active), and proper use of NRT. The first follow-up will occur 1 day before the quit date to remind participants about their quit date and provide support. The second follow-up will occur 2 weeks after the quit date to discuss progress and skills to prevent relapse and help reengage the participant in another quit attempt if they have lapsed. In our pilot trial, the two follow-ups were provided by phone. However, given the daily contact between the safety managers and CWs, we believe that a brief in-person check would be more effective and generalizable than the phone for this target population. It will also provide

more support to smokers and help change the social norm about smoking within the organization.

Group C: Participants who did not quit smoking at the 3-month follow-up after the first randomization (in Phase 1) will be re-randomized either to C or to continue in the same treatment (A 2 and B2, See study design section). Participants in group C will receive a 6-week free supply of NRT, 4 (20-min) group behavioral counseling sessions, and two brief follow-ups (in-person or by phone) (Table 5). Session 1 (2 weeks before the quit date) discusses reasons to quit, preparing to quit, coping with negative affect and job stress, and the "5 A's". Session 2 (on the quit date) focuses on short-term relapse prevention training, surviving the first few days as a nonsmoker, challenges in previous quit attempts, and managing nicotine withdrawal. In session 3 (2 weeks after the quit date), we determine if participants quit (if not, we process reasons and reset the quit date) and review motivating images. Session 4 (4 weeks after the quit date) focuses on long-term relapse (4 rules for limiting access to cigarettes, personalized relapse plan), and negative thoughts. Gumsuse and problem-solving of any adherence or side effect issues will be discussed in every session. In the first follow-up (6 weeks after the quit date) and second follow-up (8 weeks after the quit date), we will discuss progress and skills to prevent relapse and help reengage another quit attempt if they have lapsed.

All participants will receive educational information about (1) the extra risks of smoking on CWs, given their exposure to occupational hazards (e.g., injuries, and exposure to toxins), and (2) the need to consider quitting all tobacco products. Participants will be given self-help materials in their preferred language summarizing their program content.

The Intervention Fidelity

To ensure standardization of program content and delivery, we will use standardized training and treatment manuals/procedures. Safety managers will complete a checklist at the end of each session to document whether treatment components were delivered or not (we added the checklists for each program with our modifications). Participants will respond to a brief questionnaire at the 3-month follow-up in Phase 1 and 3-month follow-up in Phase 2 to assess whether key points were learned, including the techniques and information discussed in the counseling sessions. All participants will be asked about the number and content of contacts with TQL. Additionally, we will audiotape a random sample of 10% of counseling sessions for review and feedback. We will also develop a structured intervention fidelity checklist to guide the interventionists and ensure compliance with the intervention. Participants will be asked for permission to audiotape these sessions as part of the consent process.

6) **Data and Specimen Banking***

N/A

7) Data Management*

Data from the baseline assessment and survey will be collected and stored in REDCap and accessed only by approved study personnel.

8) Risks to Subjects*

The main risks to CWs' and safety managers' participation in the study are the time taken away from their work schedule and the discomfort they may feel in answering some questions about their work environment, role in the company, and other areas related to their job. To avoid coercion of CWs' and safety managers' participation in the study, we have obtained a commitment from company leaders that their participation in the study will be completely voluntary and will not impact employment status or benefits. Furthermore, participants will be assured that their data will be kept confidential and that they are free not to answer any questions.

To minimize the risk of psychological distress among CWs due to questionnaire completion, participants may skip any question that they do not wish to answer. CWs might also feel uncomfortable during receiving smoking cessation counseling from their safety manager. To minimize this risk, CWs will be informed that their participation in the study is voluntary and that they can withdraw from the study at any time. However, given the brief intensity of our intervention, and the strong relationship between safety managers and CWs, we anticipate that this risk is minimal. Safety managers are responsible for the safety and well-being of CWs, are trusted by the CWs, and have daily contact with them, which makes them optimal for the delivery of a sustainable smoking cessation treatment. Indeed, safety managers played key roles as liaisons between company leaders, researchers, and CWs in our pilot trial. They were also identified by leaders as the best people to deliver workplace tobacco treatment.

Finally, to minimize risks associated with nicotine gum use, CWs will be provided with a thorough education on the nicotine gums' proper use, indications, possible side effects, and known health conditions that preclude their use. The gums are available over the counter; thus, they do not pose a significant health risk to most people. They contain minimal amounts of nicotine (which are much less than they get from smoking) and are absent from the remaining 7,000 toxins contained in cigarettes.

Safety managers might also feel uncomfortable while delivering the smoking cessation treatment to workers and conducting behavioral counseling. To minimize this risk, in addition to the two-day in-person training in the study procedures, safety managers will be asked to complete a 3-hour self-paced tobacco treatment online via the Florida Area Health Education Centers Network (AHEC) Cessation Program. This online program provides online tobacco cessation educational modules to deliver effective evidence-based tobacco use treatment and cessation services. We will also use standardized treatment manuals and procedures. Safety managers will complete a checklist at the end of each session to document whether treatment components were delivered or not.

Additionally, we will audiotape a random sample of 10% of counseling sessions for review and feedback during the study. Finally, safety managers will have a biweekly meeting with Dr. Asfar for monitoring, case review, and problem-solving.

9) Potential Benefits to Subjects*

There are no direct benefits to participating in the study. Participants will receive help to quit smoking and improve their health.

10) Vulnerable Populations*

None

11) Setting

The two studies will take place at the Don Soffer Clinical Research Center at the University of Miami and at construction company sites.

12) Resources Available

The PI of the study, Dr. Taghrid Asfar, has many years of experience in conducting smoking cessation trials.

13) Recruitment Methods

Three levels of recruitment are planned:

1. Company leaders: We will recruit 8 companies for the study. We have secured letters of support from twelve construction companies that agreed to participate in the study (see LOS from leaders from 9 companies). We will organize a meeting with company leadership to discuss the project in detail and recruit 12-18 company leaders (≈1-2/company) who are >18 years, and involved in decision-making, to participate in the implementation process. These individuals will be contacted by our research staff to sign the consent form and be enrolled in the study.
2. Safety managers: We will recruit 20-25 (≈2/company) safety managers for the study. Twelve safety managers have already been recruited (see LOS from safety managers from 12 companies). All other safety managers in the participating companies will be invited to participate in the study. Those who are interested will be screened for eligibility, and our RAs will obtain electronic informed consent via REDCap.
3. CWs: We will recruit 608 CWs for the study. As we did in our pilot study, the safety manager who participates in the study will describe the study to CWs at the end of their regular safety morning briefings and invite them to participate. Additionally, safety managers will receive flyers and posters to display on-site. The flyers will include study details and a QR code to collect participant contact information and screen them. Those who agree to participate will meet our RAs

around the lunch truck during their lunch break to be screened and, if eligible, sign an electronic informed consent via REDCap.

14) Local Number of Subjects

We will recruit up to 12-18 company leaders (≈1-2/company), 20-25 safety managers (≈2/company), and 608 CW participants.

15) Confidentiality

This study will be anonymous, and no identifying information will be collected. Participants will be only identified in transcripts, reports, or publications with a number that is not linked to their name. To ensure confidentiality, CWs screening will be done individually one by one) using the safety manager field office. CWs who are not eligible or decline to participate will be given a list of available cessation resources.

16) Provisions to Protect the Privacy Interests of Subjects

Participants can be assured that the data they provide will only be seen by approved research staff, and they will not be identified in any publication. The Sylvester Comprehensive Cancer Center (SCCC) will monitor this clinical trial according to the Cancer Center's data and safety monitoring (DSM) plan to assure the well-being of patients enrolled on Investigator-Initiated Trials that do not have an outside monitoring review. If the PI have concerns about unexpected safety issues, the study will be stopped and will not resume until the issues are resolved. The SCCC DSM Plan to which this study is subject can also be found at www.sylvester.org.

17) Consent Process

Participants will read the electronic consent form and indicate their consent in REDCap by checking the box prior to beginning the survey. Eligible company leaders will be contacted by research staff to complete the electronic consent form and be enrolled in the study. Safety managers who are interested will be screened for eligibility and complete the electronic consent form obtained by a RA via REDCap. Construction workers who agree to participate will meet our RAs during their breakfast or lunch break to be screened, and, if eligible, sign an electronic informed consent via REDCap.
